# Mammalian keratin associated proteins (KRTAPs) subgenomes: disentangling hair diversity and adaptation to terrestrial and aquatic environments

**DOI:** 10.1186/1471-2164-15-779

**Published:** 2014-09-10

**Authors:** Imran Khan, Emanuel Maldonado, Vítor Vasconcelos, Stephen J O’Brien, Warren E Johnson, Agostinho Antunes

**Affiliations:** CIMAR/CIIMAR, Centro Interdisciplinar de Investigação Marinha e Ambiental, Universidade do Porto, Rua dos Bragas 177, 4050-123 Porto, Portugal; Departamento de Biologia, Faculdade de Ciências, Universidade do Porto, Rua do Campo Alegre, 4169-007 Porto, Portugal; Theodosius Dobzhansky Center for Genome Bioinformatics, St. Petersburg State University, St. Petersburg, 199004 Russia; Nova Southeastern University, Oceanographic Center 8000 N. Ocean Drive, Ft Lauderdale, Florida 33004 USA; Smithsonian Conservation Biology Institute, National Zoological Park, 1500 Remount Rd, Front Royal, VA 22630 USA

**Keywords:** Concerted evolution, Gene family, Keratin Associated Proteins, Keratin, Hair, Gene conversion, Recombination, Positive selection

## Abstract

**Background:**

Adaptation of mammals to terrestrial life was facilitated by the unique vertebrate trait of body hair, which occurs in a range of morphological patterns. Keratin associated proteins (KRTAPs), the major structural hair shaft proteins, are largely responsible for hair variation.

**Results:**

We exhaustively characterized the KRTAP gene family in 22 mammalian genomes, confirming the existence of 30 KRTAP subfamilies evolving at different rates with varying degrees of diversification and homogenization. Within the two major classes of KRTAPs, the high cysteine (HS) subfamily experienced strong concerted evolution, high rates of gene conversion/recombination and high GC content. In contrast, high glycine-tyrosine (HGT) KRTAPs showed evidence of positive selection and low rates of gene conversion/recombination. Species with more hair and of higher complexity tended to have more KRATP genes (gene expansion). The sloth, with long and coarse hair, had the most KRTAP genes (175 with 141 being intact). By contrast, the “hairless” dolphin had 35 KRTAPs and the highest pseudogenization rate (74% relative to the 19% mammalian average). Unique hair-related phenotypes, such as scales (armadillo) and spines (hedgehog), were correlated with changes in KRTAPs. Gene expression variation probably also influences hair diversification patterns, for example human have an identical KRTAP repertoire as apes, but much less hair.

**Conclusions:**

We hypothesize that differences in KRTAP gene repertoire and gene expression, together with distinct rates of gene conversion/recombination, pseudogenization and positive selection, are likely responsible for micro and macro-phenotypic hair diversification among mammals in response to adaptations to ecological pressures.

**Electronic supplementary material:**

The online version of this article (doi:10.1186/1471-2164-15-779) contains supplementary material, which is available to authorized users.

## Background

Terrestrial life in extant vertebrates was accompanied by the formation of diverse and rigid body coverings (scales, feathers and hairs), along with other cornified appendages (e.g. horns, hoofs, claws, nails), that evolved in response to strong selective pressures. These body coverings helped protect vertebrates and allowed them to successfully adapt to environmental pressures like heat, ultra violet radiation, water loss, and mechanical forces [1, 2]. Since keratinization helps protect the body by forming a barrier between the body and outside world, genes involved in keratinization evolve rapidly in response to changing environment (e.g., KRTAP4-5 showed evidence of positive selection in chimpanzee and hominids) [3]. Changes in gene family (gene gain, and gene loss/pseudogenization) are involved in adaptive evolution and changes in gene family size could affect expression levels [4]. In terrestrial vertebrates, the formation of hard cornified skin appendages involves interactions between fibrous (keratin) and matrix proteins (KRTAPs) [5–7]. The fibrous alpha-keratins, type I and II, appear to have evolved in stem vertebrates [8, 9] and recent studies suggest the presence of hair-specific alpha keratins orthologs in amphibians, reptiles, and birds [10–12]. However, there is no evidence of KRTAPs proteins in fishes and amphibians, suggesting that these proteins originated only after the divergence of sauropsids (sKRTAPs/beta-keratins) and mammals (mKRTAPs), leading to the formation and diversification of hard keratin appendages, feather, claw, scale in sauropsids, and hairs in mammals [5, 6, 11]. The structural and functional conservation of keratin intermediate filaments (KIFs) within mammals contrasts with the large diversity of mammalian hair phenotypes [13–15] and highlights the importance of understanding the molecular diversification of the keratin associated protein (KRATP) multigene family.

Hair is a dynamic mini-organ formed by ectodermal-mesodermal interactions [16–19] and is broadly divided into the root sheath (outer and inner), hair shaft, and matrix zone. Hair has microscopic differences (e.g., cuticular, medullar and cross section), which have long been used as forensic markers for identifying human ethnicity and classifying mammalian species [20–23]. Hair-fiber formation is a cyclical process, which involves growth (anagen), regression (catagen), and resting phases (telogen), followed by the shedding of the hair shaft. The process involves the expression of both hair keratin intermediate filament proteins and their keratin associated proteins [24–28]. This cycle is of particular importance in diverse processes such as determining hair size, shedding fur for body surface cleansing, and changing the body cover to adapt to changing environments, such as from hot summers to cold winters [29].

The current diversity of hair in extant mammals is due to innovations and changes in numerous genes and their corresponding proteins. Humans have 54 functional alpha-keratin genes comprising 28 type I and 26 type II keratins [30, 31, 13] arranged in two clusters on chromosomes 17q21.2 and 12q13.13 [32, 33], which include 11 type I and 6 type II hair keratins [34, 35]. Hair keratin types I and II undergo higher-ordered copolymerization-forming keratin intermediate filaments (KIFs) [36–39], which are embedded into a matrix formed by keratin associated proteins (KRTAPs) involved in the formation of hard cornified resilient hair shafts [40–42]. The KRTAP multigene family is divided into two broad groups, high cysteine and high glycine-tyrosine, which together comprise 30 subfamilies based on amino acid composition and phylogenetic relationships [14]. In humans, KRTAPs include approximately 100 gene members that are arranged in tandem and are clustered on chromosomes 11p15.5, 11q13.4, 17q21.2, 21q22.1, and 21q22.3 [43, 26, 44–46, 7, 25]. Given the role of the KRTAP multigene family in formation of hair morphology, we have characterized them in the genomes of 22 diverse mammalian species to provide insights on KRTAP evolution and diversification. We found contrasting KRTAP gene family repertoires among mammals, as well as differences in rates of gene expansion, contraction and pseudogenization. The two major groups of KRTAPs showed distinct evolutionary patterns with high concerted evolution influencing species-specific copy number variation and gene homogenization in high cysteine KRTAPs. In contrast, high glycine-tyrosine genes had more dynamic evolutionary patterns with less gene conversion and recombination, lower GC content, and evidence of positive selection (e.g. subfamily 20), which may also have been an important force of the evolution in subfamilies of high glycine-tyrosine.

## Results

### Genome scans

Advances in genome sequencing have made it easier to explore multigene families across different genomes. Expansion, contraction and pseudogenization, along with genomic/chromosomal organization (gene clusters) of gene families, are important mechanisms driving genome evolution and influencing fitness within lineages or species [47], as suggested by lineage- or species-specific variations in genes involved in pathogen recognition, stress response and structural proteins [48–51]. Here, we explored the KRTAP multigene family in the genome assemblies of 22 mammalian species: (1) alpaca (*Vicugna pacos*) low-coverage 2.51×, assembly, vicPac1, Jul 2008, (2) armadillo (*Dasypus novemcinctus*) low-coverage 2×, assembly, dasNov2, Jul 2008, (3) bushbaby (*Otolemur garnettii*) low-coverage 1.5×, assembly, otoGar1, May 2006, (4) cow (*Bos taurus*) coverage 7×, assembly Btau_4.0, Oct 2007, (5) dolphin (*Tursiops truncatus*) low-coverage 2.59×, assembly, turTru1, Jul 2008, (6) elephant (*Loxodonta africana*) coverage 7×, assembly, Loxafr3.0, Jul 2009, (7) gibbon (*Nomascus leucogenys*) whole genome coverage 5.6×, assembly, Nleu1.0, Jan 2010, (8) gorilla (*Gorilla gorilla*) gorGor3, Dec 2009, (9) guinea Pig (*Cavia porcellus*) high-coverage 6.79×, assembly, cavPor3, Mar 2008, (10) hedgehog (*Erinaceus europaeus*) low-coverage 1.86×, assembly, eriEur1, Jun 2006, (11) horse (*Equus caballus*) coverage 6.79×, assembly, Equ Cab 2, Sep 2007, (12) marmoset (*Callithrix jacchus*), NCBI build 1.1, (13) megabat (*Pteropus vampyrus*) low-coverage 2.63× assembly, pteVam1, Jul 2008, (14) mouse Lemur (*Microcebus murinus*) low-coverage 1.93×, assembly, micMur1, Jun 2007, (15) orangutan (*Pongo abelii*) NCBI build 1.2, (16) panda (*Ailuropoda melanoleuca*) high-coverage, assembly, ailMel1, Jul 2009, (17) pig (*Sus scrofa*) from NCBI build 3.1, high-coverage, assembly, Sscrofa10, Jun 27, 2011, (18) rabbit (*Oryctolagus cuniculus*) high-coverage, assembly, oryCun2, Nov 2009, (19) sloth (*Choloepus hoffmanni*) low-coverage 2.05×, assembly, choHof1, Sep 2008, (20) tarsier (*Tarsius syrichta*) low-coverage, 1.82× assembly, tarSyr1, Jul 2008, (21) tree shrew (*Tupaia belangeri*) low-coverage 2×, assembly, tupBel1, Jun 2006, and (22) wallaby (*Macropus eugenii*) coverage 2×, assembly, Meug_1.0, Dec 2008, available at Ensembl and NCBI websites http://www.ensembl.org/ [52, 53] and http://www.ncbi.nlm.nih.gov/ [54].

### Characterization of KRTAP gene family

**Figure 1:**
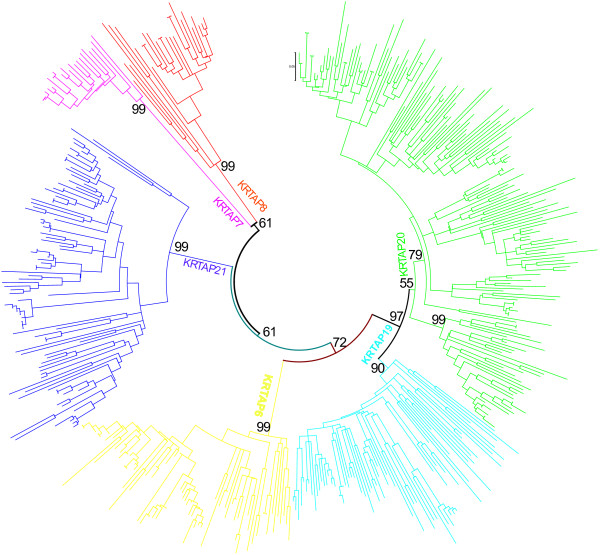
**The phylogeny of all high glycine-tyrosine gene family members of 22 mammalian genomes.** Neighbor-joining method used with P-distance and interiors branch test with 1,000 replications. The different color represents different subfamilies of high glycine-tyrosine KRTAP.

The KRTAP multigene family consists of ~100-180 gene members divided into two major classes, High Cystine (HS) and High Glycine/Tyrosine (HGT), which in turn are divided into many subfamilies with unique motifs and sequence repeats. We assigned all KRTAP multigene family members to their respective subfamilies following previously published guidelines [14]. We built species-specific phylogenetic trees to classify the gene subfamilies for each genome (Additional file 1: Figures S1-21), as well as a phylogenetic tree incorporating all members belonging to the high glycine-tyrosine KRTAP multigene family from 22 genomes (Figure 1 and Additional file 1: Figure S24). We also observed that one-to-one orthologous relationships diminished as species diverged over time (Additional file 1: Figures S22 and S23). We used the amino acid composition, unique motifs and sequence repeats, as well as blast results, to classify intact genes, partial genes and pseudogenes. The most-closely-related subfamilies are generally located in close proximity and in tandem arrangements in the genome, as are the members of the same subfamily. Due to the incomplete nature of the genomes analyzed, not all the genes may have been retrieved. Therefore, in the dolphin and other species of low genome coverage, we have assumed that missing genes may be due to low coverage that will be characterized by future research. However, we found a large number of pseudogenes in the dolphin genome compared with other low coverage genomes.

To prove the absence of KRTAP genes in high coverage genomes with intact gene clusters, we performed synteny analysis and searched for human orthologs that should be flanking the missing KRTAP genes. For example, in pig the 5’ and 3’ human orthologs flanking the KRTAP cluster 5 was missing, indicating that this region has most-likely not been sequenced and that further research and/or higher genomic coverage is needed for confirmation. We verified the synteny of conserved orthologs flanking the missing genes for the subfamily KRTAP25 in the callitrix, cow and elephant, along with KRTAP25, KRTAP19 and KRTAP29 in cavia, and KRTAP12 in rabbit.

Species-specific subfamily differences, changes in the total number of genes, functional genes, pseudogenes, amino acid content (changes in sulfur content are responsible for disulphide bonds, which provide rigidity, strength and flexibility to hair) and size polymorphism in genes within subfamilies may be responsible for the species-specific hair characteristics and the marked variability found in hair patterns among mammalian species.

### Genomic organization of the KRTAP gene family

**Figure 2:**
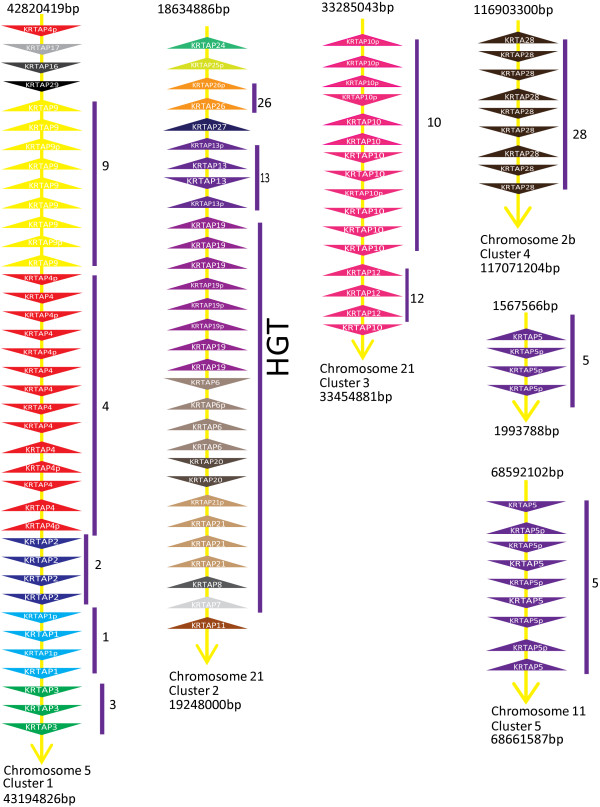
**Genomic organization of KRTAP gene family in the gorilla genome.** The KRTAP gene family is arranged in five different clusters, shown with the size in base pairs (bp) for each cluster with name of cluster and chromosome in which they are present. Each triangle represents a gene member; where p means a pseudogene, same subfamily members are shown with same colors. The triangle points the direction of transcription. The distance between the genes is not to scale.

The KRTAP gene family consists of 30 subfamilies, 24 of which are high cysteine and six are high glycine-tyrosine. The complete KRTAP gene family is arranged into five clusters at five different genomic locations (Figure 2). Each cluster contains members of one or more subfamilies arranged in a tandem array. The genomic organization of the KRTAP gene family is similar in all species studied, with only slight variations. Subfamilies KRTAP 1, 2, 3, 4, 9, 16, 17 and 29 are present in cluster one. All high glycine-tyrosine (HGT) KRTAP subfamilies, together with KRTAP 11, 13, 24-27 subfamilies, form cluster two. Subfamilies KRTAP10 and KRTAP12 form cluster three, whereas cluster four consists of subfamily KRTAP28 and cluster five of subfamily KRTAP5.

Cluster 5 shows some variation. For example in primates, KRTAP cluster 5 is divided into two paralogous gene clusters, most likely through segmental duplication, with both clusters having members of the KRTAP5 subfamily (Figure 2). In all of the other mammals studied, genes of the KRTAP5 subfamily form a single cluster. The KRTAP subfamilies that are clustered together in the genome (Figure 2) are phylogenetically closely related (Additional file 1: Figure S1) (e.g. all subfamilies of high glycine-tyrosine KRTAPs are located in close proximity in cluster 2 represented by HGT in Figure 2, which supports their functional relatedness and common ancestry arising from duplications and divergence. The conserved genomic organization of the KRTAP gene clusters over more than 166 Myr (i.e. divergence of therian from the monothermes mammals) [55] confirms the strong evolutionary constrain acting on their genomic arrangement [56]. The conserved clustering of KRTAPs seems to be related with its ordered expression in follicle [57].

### KRTAP Gene family dynamics and hair characteristics

Previously, the KRTAP gene repertoire had been assessed in eight mammalian species [14], all terrestrial species with few characteristic differences in hair phenotypes. Here we expanded on previous results by analyzing 22 additional mammalian species consisting of a much more diverse group of mammals including species from different mammalian orders with diverse hair characteristics, such as the armadillo (modified scales), hedgehog (spines), alpaca (fiber), sloth (symbionts) and dolphin (mostly hairless and aquatic), along with several more-closely related species, e.g. members of hominidae family in primates.

**Figure 3:**
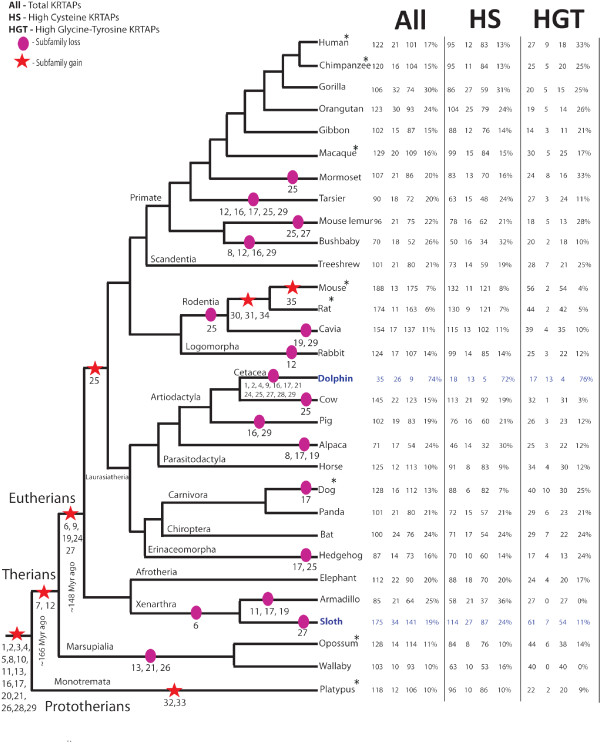
**The topological tree representing evolution of KRTAP gene family repertoires in 30 mammalian species.** Twenty-two from the present study and eight from Wu et al, 2008 [14] marked with an asterisk). Stars and circles respectively show the gain and loss of subfamilies, by numbers below.

**Figure 4:**
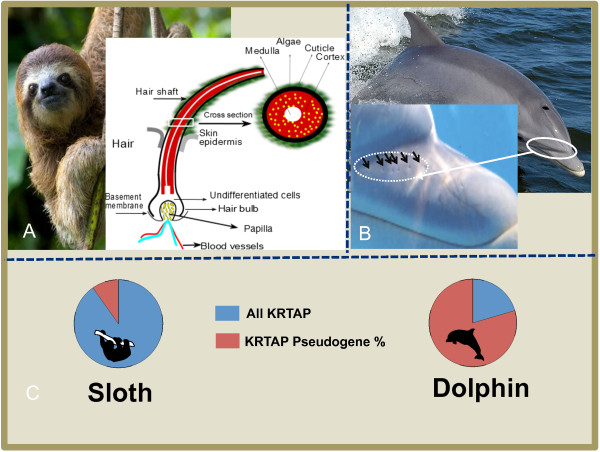
**Hair characteristic adaption in terrestrial and aquatic mammal.** Sloth an arboreal mammal with high density of hair harboring algae (image credits: firstworldfacts.com). Representation of sloth hair with algal growth and cross section of hair showing the major layers of hair shaft **(A)**. Bottlenose dolphin (image credits: Public source Dolphin NASA) with rostrum selected in circle and detailed in image with arrows point the hairless vibrissae crypts of dolphin (image credits: Élio A. Vicente, Zoomarine) **(B)**. Overall number of KRTAP genes and percentage of pseudogene present in sloth and dolphin **(C)**.

**Figure 5:**
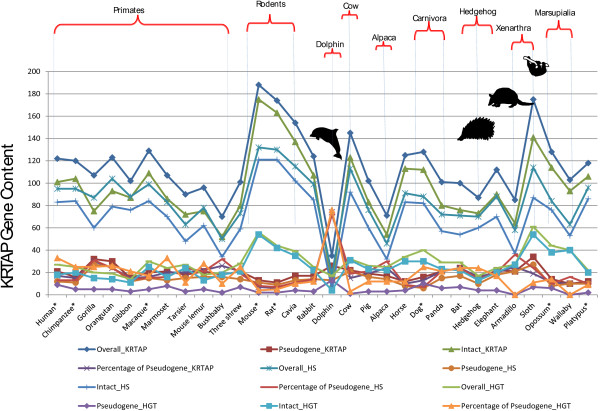
**Variation in KRTAP gene family in mammals and relation with hair characteristic features.**

We identified near complete KRTAP gene repertoires in 22 mammalian genomes, including 11 high-coverage genomes (Table 1, Figure 3, and Additional file 2). Our findings suggest that the most recent common ancestor of mammals is supposed to have had 53% (16 of 30) of the known KRTAP subfamilies (1-5, 8, 10, 11, 13, 16, 17, 20, 21, 26, 28, and 29) (Figure 3). Extant monotremes (Platypus) and marsupials (Opossum and Wallaby), have slightly different subfamilies representation (60%, 18 of 30 subfamilies, and 50%, 15 of 30, respectively), while eutherians have up to 93% (28 of 30) of the KRTAP subfamilies. This shows that the diversification of the KRTAP gene family occurred early in mammalian evolution, likely starting after the split of sauropsids (leading to birds and reptiles) and synapsids (leading to mammals-like reptiles) around 350 Myr ago [58, 55]. Sauropsids developed KRTAPs (the beta keratins) in hard appendages like feathers, beaks, scales and claw, etc., and synapsids developed mKRTAPs present in hair, nails, hoofs, claws, etc. The presence of glycine rich proteins such as HGT in mammals and HGP (high glycine proline) in reptiles and birds is evidence of their radiation from a common ancestor [5] and suggest that these changes may have contributed to the successful radiation of mammals, reptiles and birds. Further expansion and diversification of the KRTAP gene family, favored by high rates of concerted evolution in HS-KRTAPs and positive selection in HGT-KRTAPs, led to the species-specific hair characteristics observed in extant mammals. Additional analyses of sauropsid and the mammalian KRTAPs are likely to reveal insights into the patterns of adaptive radiation present in extant reptilian, birds and mammalian KRTAPs. Subfamilies 7 and 12 first appear in therian mammals after their divergence from monothremes around 166 Myr ago [55]. Subfamilies 6, 9, 19, 24, and 27 are specific to placental mammals (eutherians), and thus appeared after their divergence from marsupials around 148 Myr ago [55]. Subfamily 25 is absent in Afrotheria and Xenarthra, which suggests an origin within placental mammals only after the divergence from the atlantogenata clade (Figure 3 and Table 1). Monotremes and marsupials lack subfamily 9, which we observed to have expanded dramatically in the basal placental mammal xenartha (sloth) to 50 members. We noted that the KRTAP gene family shows species-specific variation as expected due to concerted evolution, and some of the subfamilies are restricted to particular species, e.g. subfamilies 30, 31, and 34 are present only in mouse and rat, subfamily 35 in mouse, and subfamilies 32 and 33 in platypus [14] (Figure 3 and Table 1). We also observed remarkable differences among these KRTAP genes (Table 1, Figure 3 and Additional file 2), including a dramatic gene expansion with 175 members (50 in subfamily 9 and 37 genes in subfamily 20, respectively) in sloth (*Choloepus hoffmanni)*, a nocturnal hairy mammal with long, coarse and shaggy fur that serves as a host for different microorganism [59] (Table 1, Figures 3 and 4). Similarly, we found gene expansion in subfamily 20 (27 genes copies) in the rodent Guinea Pig (*Cavia porcellus*), and 38 genes copies in the marsupial Wallaby (*Macropus eugenii*). Subfamily 28 has expanded in Rabbit (*Oryctolagus cuniculus*) (23 genes copies), which belongs to order lagomorpha. We typically observed functional genes in (HS-KRTAPs) subfamilies 11, 16, 17, 24-27 and 29 varying from a minimum of one to a maximum of three. The subfamilies 11, 16, 17 and 25 have a maximum of one functional gene member, subfamilies 24 and 29 have a maximum of two functional genes (present in orangutan and cow, respectively), and subfamily 26 has a maximum of three members (present in sloth and elephant). Subfamily 7, belonging to the high glycine-tyrosine group, has a maximum of one functional gene member (Table 1). We found that closely related species, e.g. among Hominidae family (Human, chimpanzee, gorilla and orangutan), have very similar gene repertoires with only slight differences (e.g. Humans have highest HGT pseudogenes; Figure 3). We also observed the apparent reduction in the KRTAP gene repertoire in alpaca (fibre), armadillo (modified scales), hedgehog (spines), and dolphin (mostly hairless and aquatic) (Figure 3, Figure 5), probably due to the replacement or modification of hair function or to extensive specialization and subsequent selection and pseudogenization.

**Table 1:** **Number of KRTAP gene present in each subfamily in twenty-two mammalian species**

KRTAP		Gorilla	Pongo	Gibbon	Calllitrix	Tarsius	Mouse lemur	Otolemur	Treeshrew	Cavia	Rabbit	Dolphin	Cow	Pig	Alpaca	Horse	Panda	Bat	Hedgehog	Elephant	Armadillo	Sloth	Wallaby
HS-KRTAP	KRTAP1	4(2)	4(0)	3(0)	4(1)	4(0)	4(0)	4(0)	2(1)	4(0)	4(0)	0(0)	4(0)	4(1)	5(1)	4(0)	3(0)	4(1)	4(1)	4(0)	2(0)	4(1)	4(1)
	KRTAP2	4(0)	3(0)	4(0)	4(0)	3(0)	4(0)	2(0)	5(1)	4(0)	4(0)	0(0)	4(0)	4(0)	3(0)	4(0)	5(1)	3(0)	4(1)	4(0)	4(0)	3(0)	5(0)
	KRTAP3	3(0)	6(0)	4(1)	4(0)	3(0)	2(0)	4(1)	2(1)	4(0)	4(0)	2(2)	4(0)	4(0)	5(2)	4(0)	4(1)	4(0)	4(0)	4(1)	3(1)	4(0)	4(0)
	KRTAP4	15(6)	15(4)	10(2)	8(1)	8(1)	9(2)	14(3)	12(1)	16(1)	14(1)	0(0)	16(5)	7(0)	7(3)	12(0)	4(1)	6(0)	8(1)	13(4)	9(3)	8(0)	17(4)
	KRTAP5	13(8)	14(6)	15(3)	19(5)	16(8)	8(4)	13(2)	11(4)	18(5)	18(1)	1(1)	18(5)	0(0)	2(2)	11(2)	13(3)	6(4)	5(0)	12(4)	8(7)	8(1)	7(1)
	KRTAP9	9(2)	12(2)	9(1)	5(0)	6(1)	4(1)	8(0)	5(0)	12(2)	6(1)	0(0)	19(3)	5(0)	4(1)	8(2)	2(0)	9(3)	9(1)	4(0)	7(2)	50(5)	0(0)
	KRTAP10	13(5)	17(2)	13(0)	13(2)	8(2)	5(1)	7(1)	10(2)	12(1)	10(2)	2(2)	15(3)	14(1)	1(1)	11(0)	8(1)	14(5)	1(1)	8(1)	3(1)	1(0)	5(3)
	KRTAP11	1(0)	1(0)	1(0)	1(0)	1(0)	1(0)	1(0)	1(0)	1(0)	1(0)	1(1)	1(0)	1(0)	1(0)	1(0)	1(0)	1(0)	1(1)	1(0)	0(0)	1(0)	1(0)
	KRTAP12	3(0)	4(2)	2(0)	3(2)	0(0)	0(0)	3(1)	1(0)	12(1)	0(0)	1(0)	6(0)	4(0)	1(0)	10(1)	5(1)	1(0)	10(1)	11(2)	1(0)	5(2)	7(0)
	KRTAP13	4(2)	8(4)	7(3)	6(1)	4(2)	4(2)	8(0)	6(1)	8(2)	8(3)	10(6)	8(3)	14(10)	8(0)	10(2)	8(1)	12(3)	7(1)	11(5)	9(5)	16(13)	0(0)
	KRTAP16	1(0)	2(1)	1(0)	1(0)	0(0)	0(0)	1(0)	1(0)	1(0)	1(0)	0(0)	1(0)	0(0)	1(0)	1(0)	1(0)	1(0)	2(1)	1(0)	1(0)	2(1)	1(0)
	KRTAP17	1(0)	1(0)	1(0)	1(0)	0(0)	1(0)	1(1)	1(0)	1(0)	1(0)	0(0)	1(0)	1(0)	0(0)	1(0)	1(0)	1(0)	0(0)	1(0)	0(0)	1(0)	1(0)
	KRTAP24	1(0)	2(0)	1(0)	1(0)	1(0)	1(0)	1(1)	1(0)	1(0)	1(0)	0(0)	1(0)	1(1)	1(0)	1(0)	1(0)	1(0)	1(0)	1(0)	1(0)	1(0)	0(0)
	KRTAP25	1(1)	1(1)	1(0)	0(0)	0(0)	1(1)	0(0)	1(1)	0(0)	1(1)	0(0)	0(0)	1(1)	1(1)	1(0)	1(0)	1(1)	0(0)	0(0)	0(0)	0(0)	0(0)
	KRTAP26	2(1)	1(0)	2(0)	1(0)	1(0)	1(1)	1(1)	1(0)	1(0)	1(0)	1(1)	2(0)	1(0)	1(0)	1(0)	1(0)	1(0)	1(0)	3(0)	1(0)	4(1)	0(0)
	KRTAP27	1(0)	1(0)	1(0)	1(0)	1(0)	1(1)	0(0)	1(0)	1(0)	1(0)	0(0)	1(0)	1(0)	1(0)	1(0)	1(0)	1(0)	1(1)	1(0)	1(0)	0(0)	0(0)
	KRTAP28	9(0)	11(1)	12(2)	10(1)	7(1)	4(3)	9(4)	11(3)	19(1)	23(5)	0(0)	10(2)	14(2)	2(2)	9(1)	12(6)	4(0)	11(0)	8(1)	7(2)	5(2)	10(1)
	KRTAP29	1(0)	1(1)	1(0)	1(0)	0(0)	0(0)	1(1)	1(0)	0(0)	1(0)	0(0)	2(0)	0(0)	2(2)	1(0)	1(0)	1(0)	1(0)	1(0)	1(0)	1(1)	1(0)
	KRTAP30	0	0	0	0	0	0	0	0	0	0	0	0	0	0	0	0	0	0	0	0	0	0
	KRTAP31	0	0	0	0	0	0	0	0	0	0	0	0	0	0	0	0	0	0	0	0	0	0
	KRTAP32	0	0	0	0	0	0	0	0	0	0	0	0	0	0	0	0	0	0	0	0	0	0
	KRTAP33	0	0	0	0	0	0	0	0	0	0	0	0	0	0	0	0	0	0	0	0	0	0
	KRTAP34	0	0	0	0	0	0	0	0	0	0	0	0	0	0	0	0	0	0	0	0	0	0
	KRTAP35	0	0	0	0	0	0	0	0	0	0	0	0	0	0	0	0	0	0	0	0	0	0
	**TOTAL-HS**	**86(27)**	**104(25)**	**88(12)**	**83(13)**	**63(15)**	**78(16)**	**50(16)**	**73(14)**	**115(13)**	**99(14)**	**18(13)**	**113(21)**	**76(16)**	**46(14)**	**91(8)**	**72(15)**	**71(17)**	**70(10)**	**88(18)**	**58(21)**	**114(27)**	**63(10)**
HGT-KRTAP	KRTAP6	4(1)	3(1)	3(0)	3(0)	8(0)	4(0)	4(1)	5(0)	7(1)	9(1)	1(1)	5(0)	4(0)	9(0)	4(0)	7(2)	6(0)	4(1)	5(1)	0(0)	0(0)	0(0)
	KRTAP7	1(0)	1(0)	1(0)	1(0)	1(0)	2(0)	2(0)	1(0)	1(0)	1(0)	1(1)	1(0)	1(0)	1(0)	1(0)	1(0)	1(0)	1(0)	1(1)	1(0)	1(0)	1(0)
	KRTAP8	1(0)	1(0)	1(0)	1(0)	2(0)	0(0)	0(0)	1(1)	1(0)	1(0)	3(0)	2(0)	4(1)	0(0)	3(1)	1(0)	1(0)	1(0)	1(0)	1(0)	4(2)	1(0)
	KRTAP19	8(3)	9(4)	5(3)	9(5)	10(3)	5(0)	6(0)	9(2)	0(0)	2(0)	6(5)	8(1)	3(2)	0(0)	8(2)	8(3)	7(2)	4(0)	6(1)	0(0)	7(1)	0(0)
	KRTAP20	2(0)	2(0)	1(0)	2(1)	4(0)	6(1)	5(1)	10(3)	27(3)	8(1)	6(6)	5(0)	5(0)	12(2)	12(0)	4(0)	6(3)	2(2)	6(1)	12(0)	37(3)	38(0)
	KRTAP21	4(1)	3(0)	3(0)	8(2)	2(0)	3(1)	3(1)	2(1)	3(0)	4(1)	0(0)	11(0)	9(0)	3(1)	6(1)	8(1)	8(2)	5(1)	5(0)	13(0)	12(1)	0(0)
	**TOTAL-HGT**	**20(5)**	**19(5)**	**14(3)**	**24(8)**	**27(3)**	**18(5)**	**20(2)**	**28(7)**	**39(4)**	**25(3)**	**17(13)**	**32(1)**	**26(3)**	**25(3)**	**34(4)**	**29(6)**	**29(7)**	**17(4)**	**24(4)**	**27(0)**	**61(7)**	**40(0)**
	**ALL-KRTAP**	**106(32)**	**123(30)**	**102(15)**	**107(21)**	**90(18)**	**96(21)**	**70(18)**	**101(21)**	**154(17)**	**124(17)**	**35(26)**	**145(22)**	**102(19)**	**71(17)**	**125(12)**	**101(21)**	**100(24)**	**87(14)**	**112(22)**	**85(21)**	**175(34)**	**103(10)**

Number of pesudogene is represented in parenthesis.

For example, we observed high rates of pseudogenization (Figures 3, 4 and 5 and Table 1) (74% compared to the mammalian average of 19%) and only nine intact genes in the dolphin (*Tursiops truncatus*). This aquatic mammal is almost hairless, with only a few hairs (bristles) on the upper lip of the rostrum, which are shed soon after birth, leaving hairless pits on the rostrum of adults that have specialized sensory function [60–65] (Figure 4). The epidermal surface also undergoes high proliferation and sloughing of epidermis cells in order to maintain a smooth skin, a major advantage for swimming [66, 67].

### Concerted evolution, GC bias and sequence divergence

Tandemly arranged gene members of multigene families often show more similarity among each other than with their counterpart’s orthologs in other species, which suggest that they evolved in similar or concerted fashion. This would further lead to species-specific variation, as observed in KRTAP gene family. Two mechanisms play an important role in concerted evolution. Recombination increases the copy number of gene by providing raw material for further functional innovations and diversification, and gene conversion, which principally homogenizes genes, can help insure the rapid synthesis of a gene product (protein) that may be required during a precise stage of cell cycle [68]. Gene conversion also decreases the evolutionary distance among paralogous members and shifts the substitutions from weak (A or T) to strong (G or C) by increasing GC content through biased gene conversion (gBGC) [69–72]. The negative correlation between evolutionary distance calculated by synonymous substitution rates and GC content provides the level of divergence between the members of a subfamily [73].

Using Geneconv [74] and RDP3 [75] we found higher rates of gene conversion and recombination events in the high cysteine KRTAPs compared with the high glycine-tyrosine KRTAP genes (Additional files 3 and 4). KRTAP subfamilies also displayed different rates of gene conversion in different species. For example, in gorilla we found 44 gene pairs of the KRTAP10 under gene conversion, compared to only 17 gene pairs found for this subfamily in gibbon (Additional file 3). The high level of gene conversion also reduces orthologous relationship between genes of two different species.

**Figure 6:**
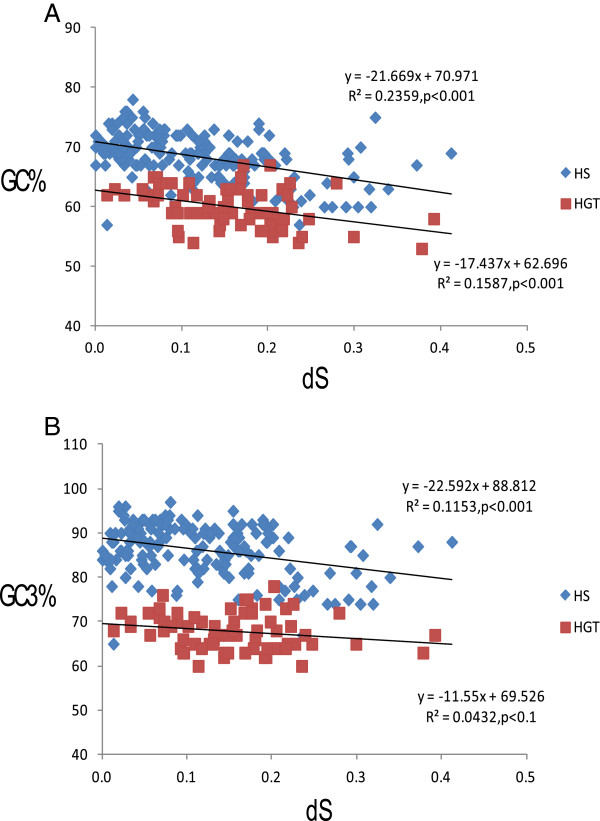
**GC-content dynamics.** Figure legend text GC-biased gene conversion (gBGC) and evolutionary distance between the KRTAP genes, shown by the correlation between the synonymous substitution rates (dS) and GC content (GC%) among paralogous members of each subfamily **(A)** and third codon GC content (GC3%) **(B)**. Negative correlation points towards the gene conversion. High cyteine KRTAP (HS) and high glycine-tyrosine KRTAP (HGT) are represented by blue and red squares respectively. The linear regression is shown.

Sequences with higher synonymous substitution rates (dS) had higher overall GC content (GC%) and third-codon GC content (GC3%), and lower synonymous substitution rates in the high-cysteine genes than the high glycine-tyrosine genes. The negative correlation between GC content and synonymous substitution rate (dS) is consistent with the higher rates of concerted evolution observed in high cysteines (Figure 6A and B). The high GC content in the HS –KRTAP gene family compared with the HGT-KRTAP could be a consequence of the high number of gene conversion events.

### Adaptive evolution

Gene expansion provides the essential raw material for the positive selection to act [76], which in turn accelerates the diversification of duplicated copies by increasing the number of nonsynonymous substitutions (dN) relative to the synonymous substitutions (dS) through positive selection (dN/dS > 1). The PAML package [77] was used to identify signatures of positive selection. Specifically, we used likelihood ratio test for positive selection [78, 79] to test site-specific models comparing twice the difference in log-likelihood between two models to chi square distribution with two degrees of freedom. For expanded subfamilies, such as in the case of the KRTAP20 in wallaby with 38 members, we tested if this species-specific expansion has been influenced by adaptive evolution. We tested two nested pairs of site-specific models (M1a vs. M2a and M7 vs. M8), where M1a and M7 states no positive selection (ω ≤ 1) and M2a and M8 states positive selection (ω ≥ 1). In both cases the likelihood of positive selection was significantly higher (p < 0.0001), retrieving similar sites under positive selection. The likelihood ratio test is a conservative approach, which can be biased by false positives in the presence of high recombination rates [80]. Thus, we evaluated the possibility of gene conversion/recombination in the KRTAP20 subfamily that has expanded dramatically in the wallaby genome, but did not detect such evidence. The results of positive selection tests are shown in Table 2. The positive selection acting of KRTAP probably favored the diversification and adaptation to different environment.

**Table 2:** **Likelihood ratio test for PAML site models within Wallaby**

Model	Parameters	lnL	2ΔlnL (LRT)
M0	ω: = 0.65464	-1673.250363	NA
M1a	p_0_: 0.57003 p_1_: 0.42997	-1576.668729	
	ω0: 0.09616 ω1: 1.00000		
M2a	p_0_: 0.49130 p_1_: 0.31322 p_2_: 0.19547	-1556.441999	M1a vs. M2a 40.45346 (p = 1.643E-09)
	ω_0_: 0.10488 ω_1_: 1.00000 ω_2_: 3.48389		
M7	p = 0.33061 q = 0.37447	-1581.277897	
M8	p_0_ = 0.74935 p = 0.55372 q = 1.09725	-1560.301816	M7 vs. M8 41.952162 (p = 7.765E-10 )
	(p_1_ = 0.25065) ω_1_ = 2.75722		

### Differential evolution of the HS and HGT KRTAPs

**Figure 7:**
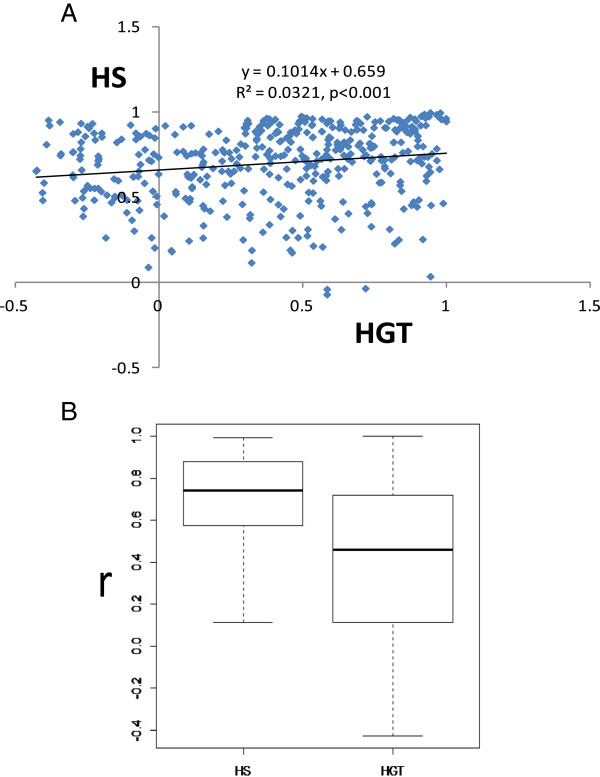
**Pearson correlation coefficients (r) show the evolutionary differentiation of KRTAP genes.** Pearson correlation coefficients (r) values of the high cysteine and high glycine-tyrosine KRTAP are positively correlated. The linear regression is shown. **(A)** The boxplot for Pearson correlation coefficients (r) of gene numbers of each subfamily between species shows, high cysteine KRTAP genes have higher correlation coefficient than high glycine-tyrosine KRTAP genes **(B)**.

The KRTAP multigene family has experienced dynamic evolution and diversification within and among genomes as observed in the 30 diverse subfamilies of high cysteine and high glycine-tyrosine subfamilies. These two groups have evolved differently, with the high cysteine group showing high rates of gene conversion within subfamilies, with some exhibiting characteristic differences in copy number, while others have been more conserved. This may be an adaptive mechanism promoting a high order of amplification of similar copies to meet the high demand for the structural proteins required to adapt to changing environmental conditions (e.g. sloth have extensive hairs that can harbor symbiotic microorganism communities while the dolphin is “hairless” in response to a more-predictable and constant environment and to create less resistance when swimming). We also compared the differential evolutionary patterns between high cysteine and high glycine–tyrosine genes using the Pearson correlation coefficient for the number of genes in each subfamily between species. The coefficient value for high cysteine is significantly higher than for high glycine-tyrosine (Figure 7A) and the coefficient values for the two are positively correlated (p < 0.001) (Figure 7B). The high GC content and negative correlation between GC content and synonymous substitution rates also support the higher rates of gene conversion observed in high cysteine genes relative to high glycine-tyrosine KRTAPs, suggesting that high cysteine are under high rates of concerted evolution promoted by gene conversion and recombination events (see Additional files 3 and 4). By contrast, HGT-KRTAP had a more-dynamic evolutionary pattern, with less evidence of gene conversion or recombination, but with signatures of positive selection.

### Size Polymorphism and amino acid composition affects KRTAP matrix formation and interactions with hair KIFs

The KRTAP family is widely grouped into three major categories based on amino acid composition: (i) high sulfur (<30% cysteine content), including subfamilies 1, 2, 3, 10-13, 16, 24-27, 29, 31, 34 and 35; (ii) ultrahigh sulfur (>30% cysteine content), including subfamilies 4, 5, 9, 17, 28, 30, 32 and 33 subfamilies; and (iii) high glycine/tyrosine, including subfamilies 6, 7, 8, 19, 20 and 21. The amino acid composition is shown in Table 3. Subfamily gene members also showed size polymorphism [81–83] mostly due to cysteine-rich repeats, which create difference in cysteine content. Cysteine is important for the formation of strong disulphide bonds. Thus, changes in cysteine composition can result in differential interaction among KRTAPs and between KIFs and KRATPs leading to combinatorial complexity and thereby creating morphological differences in hair fiber strength, rigidity and flexibility [40].

**Table 3:** **Amino acid composition of KRTAPs subfamily genes in mammals**

KRTAP	Subfamily	Cysteine	Glycine	Leucine	Proline	Glutamine	Serine	Threonine	Tyrosine
**HS-KRTAP**	KRTAP1	25.98	9.68	1.83	9.26	7.16	13.89	9.41	1.70
	KRTAP2	28.83	4.35	1.59	14.50	5.29	9.17	9.28	0.52
	KRTAP3	19.65	3.92	8.05	15.43	3.12	8.51	10.97	1.29
	KRTAP4	34.84	3.29	1.15	9.87	6.14	17.60	7.19	0.72
	KRTAP5	33.67	23.00	0.35	5.02	3.74	20.58	0.28	0.36
	KRTAP9	35.30	3.57	1.17	11.64	7.41	12.57	12.79	1.81
	KRTAP10	25.92	1.95	3.66	13.79	5.85	18.86	4.85	0.73
	KRTAP11	12.84	6.86	3.79	8.25	7.16	16.22	10.81	2.64
	KRTAP12	22.54	4.26	4.15	13.85	6.24	17.94	4.27	1.11
	KRTAP13	11.11	10.67	6.62	7.25	3.83	21.93	5.99	7.71
	KRTAP16	19.24	1.89	2.54	14.12	5.33	17.40	5.94	2.24
	KRTAP17	36.09	28.93	0.00	5.29	3.73	10.37	2.83	0.15
	KRTAP24	9.73	5.89	7.90	9.49	4.16	17.18	6.87	6.55
	KRTAP25	8.17	3.92	6.86	8.82	8.17	16.99	1.96	7.52
	KRTAP26	10.38	6.58	9.55	11.13	4.08	18.98	6.24	4.36
	KRTAP27	8.87	4.33	6.69	7.97	8.32	17.15	7.90	2.04
	KRTAP28	38.43	30.92	0.04	1.78	4.19	7.71	2.68	1.20
	KRTAP29	16.25	6.13	3.74	11.10	7.96	16.66	6.92	2.29
**HGT-KRTAP**	KRTAP6	14.37	39.89	5.40	0.11	0.02	6.96	0.16	22.07
	KRTAP7	7.82	20.26	5.99	6.85	0.29	11.93	5.65	11.76
	KRTAP8	6.15	22.47	4.52	6.64	0.14	8.83	2.69	19.15
	KRTAP19	8.07	35.88	4.51	1.53	0.10	10.39	0.33	18.18
	KRTAP20	10.62	32.08	4.97	2.22	0.13	7.01	0.54	25.05
	KRTAP21	17.88	34.32	0.76	1.76	0.16	11.50	1.77	21.10

Average percentage of eight amino acids.

## Discussion

Gene families are formed by gene duplication, a process that provides important raw material for functional innovation and adaptive selection. Gene families vary in size from a few to thousands of gene members, which makes it difficult to identify and characterize them without sufficient genome sequences. The genome sequencing projects have made it possible to explore complex gene families involved in different phenotypes. Here we explored the mammal-specific KRTAP gene family, which is the major constituent of the hair proteome and plays a primary role in hair formation and thus long been associated with phenotypic differences in hair and wool. This study assessed patterns of variation using comparative genomic approaches in the KRTAP gene family. Our study used 22 diverse mammalian genomes that encompassed closely related species, e.g. family Hominidae of primates, comparing apes with dense hair cover with human with much less hair cover, along with species with diverse hair related characteristic, e.g. alpaca (fibre), armadillo (modified scales), hedgehog (spines), sloth (hosting hair symbionts) and dolphin (mostly hairless and aquatic), to obtain greater insights into the KRTAP gene family evolution relative to mammalian hair and phenotypic variations.

We found high molecular diversity within the KRTAP gene family, with 30 subfamilies (24 belonging to high cysteine and six belonging to high glycine-tyrosine KRTAP) (Additional file 2 and Table 1) and approximately 100-180 KRTAP gene members, which are arranged in five clusters at five different chromosome locations in a genome (Figure 2). Most KRTAP subfamilies are found in all mammalian orders, with variations in expansion, contraction, presence/absence, different rates of pseudogenization and sequence variation (length polymorphisms and amino acid changes). For example, we found species-specific differences in the size and compositions of some subfamilies (e.g. subfamilies 4, 5 and 9) probably caused by unequal crossing over accompanied with high GC content. Moreover, we also found lineage-specific trends, such as in marsupials, where both wallaby and opossum lacked subfamilies 13, 21 and 26 and showed expansion of the KRTAP20 subfamily, which is under positive selection (Table 2). However, highly conserved sequences and the maintenance of the same number of members in subfamilies 1, 2, and 3 suggests that high rates of gene conversion maintain homogeneity and with evolution occurring through a process of punctuated equilibrium [14, 84, 85]. Similarly, the conserved synteny of KRTAP gene clusters shows that there are also strong constraints acting on this gene family and supports the important role of KRTAP gene family in shaping hair characteristics.

Together with the high molecular diversity of the KTRAP gene family observed in our study, considerable intraspecific diversification has been reported earlier with copy number variations [86–91] in ethnic human populations and allelic variations in sheep. Such sequence polymorphism found in KRTAP gene members may influence its expression, protein structure, and/or post-translation modifications and consequently effect wool/hair fibre structure and wool/hair quality traits [92, 23, 93]. For example, evidence of linkage reported between KRTAP6-8 and wool fiber diameter (quantitative trait) in sheep [94] may be related with similar characteristics in alpaca (fiber), which has one of the largest number of KRTAP6 genes (n = 9) in mammals. Further exploration of KRATP gene family in sheep could help shed light on the improvement of hair/wool traits [57, 95, 94, 96].

Interestingly, we found differences in KRTAP gene repertoire related with hair features. A very expanded KRTAP gene family repertoire (175 total genes and 141 intact genes) was found in sloth, which has long, dense and coarse body hair cover, which also serves as a host for symbiotic microorganisms (e.g., cyanobacteria) in this arboreal mammal. By contrast, we have detected a reduced number of functional KRTAP genes and high percentage of KRTAP pseudogenes (74%) in dolphin (aquatic mammal), highlighting the much lower KRTAP gene requirement in this smooth-skinned species that only has a few hairs (bristle) at the rostrum. These are lost soon after birth and in adults the hairless pits are adapted for sensory functions (Figure 4). This example illustrates the adaptive potential of hair follicles to diversify into more specialized sensory organs.

We also observed that several unique hair-related phenotypes in some of the species, such as scales in armadillo, fiber in alpaca and spines in hedgehog, are linked with an inverse correlation between the number of intact KRTAP genes and the number of pseudogenes. Armadillo and hedgehog have specialized hair features, where the pattern in alpaca could be due to inbreeding and genome homogenization during domestication. The “hairless” dolphin had a large number of KRTAP pseudogenes relative to intact genes (Figure 5). In contrast, the sloth showed a high positive correlation with intact KRTAP genes, suggesting that changes in KRTAP can be related to morphological diversity of hair phenotypes (Figure 5). In contrast, we did not find any correlation between the comparatively hairless human and other primates, which favors the hypothesis, that diversification of keratinization structures in mammals may be collectively explained by KRTAP gene number variation together with other biological mechanisms, such as gene expression variation in KRTAPs (which can be further influenced by KRTAP genes polymorphism).

We suggest that the diverse repertoire and variability in KRTAPs (at gene, family and genome level) provides extraordinary combinatorial complexity [97] for interaction between KRTAPs and Keratin intermediate filaments, resulting in a rich diversity of pathways for evolutionary change, which together with differences in higher order expression of KRTAP genes results in the diverse hair morphological characteristic visible in extant mammals. Overall, we conclude that KRTAPs play an important role in evolution and diversification of hair character across mammals and are responsible for unique features of hair.

## Conclusions

The present study explored KRTAP gene family evolution in various mammalian species inhabiting diverse terrestrial and aquatic environments. The two groups of the KRTAP gene family, high cysteine and high glycine-tyrosine KRTAP genes, have evolve differently, resulting in species-specific diversification of this multi-gene family and leading wide morphological diversity in hair characteristics in extant mammals. We conclude that differences in KRTAP gene family repertoires, together with changes in expression patterns, are responsible for shaping unique hair characteristics in diverse mammalian species. These differences are more pronounced between aquatic and terrestrial species and demonstrate the important adaptive role of hairs in terrestrial colonization and the radiation of mammals from water to land. Future studies comparing the KRTAP repertoire in key model organisms, e.g. Alpaca and sheep, may provide insights to understanding the role of KRTAP gene variations in hair fibre traits and its use in textile industry.

## Methods

### Gene identification

All KRTAP genes are relatively small (ca. 1 kb) and generally have single exon [83]. Some KRTAP genes appear to possess small introns. However these are similar to repeat regions present in the gene [98] and can be translated in-frame with the coding exon, leading to the conclusion that all KRTAP are intron-less [14, 99]. The presence of KRTAP gene clusters in mammalian genomes makes it easy to identify and fully characterize the gene family in genomes with high coverage, but in low coverage genomes it requires much more manual inspection and in-depth screening to insure an almost complete or maximum possible repertoire of non-redundant KRTAP genes (Additional file 2). In order to identify the complete gene repertoire in the KRTAP gene family, all previously annotated gene sequences were taken and used as query in blast searches against the genomes from Ensembl http://www.ensembl.org/Multi/Tools/Blast and NCBI genome database http://www.ncbi.nlm.nih.gov/blast/Blast.cgi using BLASTN algorithm [100] and E-value cut-off of 10. We retrieved multiple hits for each query and selected all the non-redundant hits by extending 500 bp at both 5’ and 3’ ends. Non-redundant hits, which were seen to be clustered in the same region (chromosome, contig, genescaffold, scaffold, supercontig) were merged together to form a single extended common DNA fragment, bearing all these hits and the ends of this fragment were further extended to maximum 0.3 Mbp were ever possible. Finally all the hits were used to identify and annotate KRTAP gene using program BLAST 2 Sequences [101] TFASTX and TFASTY incorporated in Fasta programs [102] and ORF finder from NCBI http://www.ncbi.nlm.nih.gov/gorf/gorf.html and Mobyle [103]. The identified gene were blast searched against non redundant NBCI blast database, all best hits which resulted in KRTAP or KRTAP like sequences were finally taken as KRTAP genes. The KRTAP genes were further classified into intact/complete genes, partial genes and pseudogene with interrupting frame-shift mutations and/or stop codons.

### Phylogenetic analysis

We employed phylogenic tree building method to further classify the identified KRTAP gene repertoire to their respective subfamilies. For each species the intact genes were used for building phylogentic tree. All intact KRTAP genes were translated to amino-acid and aligned using ClustalW incorporated in MEGA4.0 [104, 105] with Blosum protein weight matrix the manual adjustments were done when ever needed to correct the final alignment. This final protein sequence alignment was used to build the KRTAP gene tree with the Neighbour-Joining method with P-distance and the interior branch test evaluated with 1,000 replications [106] (Additional file 1: Figures S1-24). We make use of unique motifs and repeat sequence structure present in KRTAP subfamilies along with phylogeny and blast results to further help identify and classify partial and pseudogenes to the respective subfamilies.

### Gene conversion and Recombination study

We used the program Geneconv http://www.math.wustl.edu/~sawyer/geneconv/ [74] to detect statistically significant events of sequence homogenization on paralogs using Global Bonferroni corrected P values. The lower P values indicate greater support for gene conversion. The multiple sequence alignment of protein were back translated and used as input for the Geneconv to give both global and pairwise fragments involved in gene conversion. We also used the RDP3 software [75] to detect recombination events using RDP, Bootscan, MaxChi and Chimaera with 1,000 permutations and cutoff p value of 0.01 employing Bonferroni correction.

The evolutionary distance between genes can be calculated with synonymous substitution, which are immune to selection and are not decreased by negative selection [50]. The sequence divergence was estimated using approximate synonymous substitution rates (dS) implemented in MEGA using modified Nei-Gojobori (P-distance) method with transition/transversion ratio of 2. GC content was estimated using MEGA5.0 [107]. More than two sequences are needed to detect the signals of recombination therefore subfamilies having more than three genes were used for studies of gene conversion (Additional files 3 and 4).

### Statistical analysis

In order to study the differential evolutionary pattern of high cysteine and high glycine-tyrosine KRTAP genes, we compared the pairwise-pearson correction coefficient (Figure 7) of the number of genes present in each subfamily (Table 1). We also compared the correlation between GC content (GC% and GC3%) and synonymous substitution rates (Figure 6) using the Nei-Gojobori (P-distance) method with transition/transversion ratio of 2 in MEGA4.0 [95].

### Availability of supporting data

All the supporting data are included as additional files.

## Electronic supplementary material

Additional file 1: Figure S1-S24: The phylogeny of high cysteine KRTAP genes in 22 mammalian species. Neighbor-joining method with P-distance and interiors branch test with 1,000 replications (shown on the branches) was employed to build the trees. Figures S23 and S24 shows loss of, one to one orthologous relationship between two species due to concerted evolution. The KRTAP members are labeled with species abbreviation, Gene ID and KRTAP subfamily (Additional file 2) Figure S1-21 are in order, Gorilla, Pongo, Gibbon, Mormoset, Tarsies, Mouse lemur, Bushbaby, Treeshrew, Cavia, rabbit, Cow, Pig, Alpaca, Horse, Panda, Bat, Hedgehog, Elephant, Armadillo, Sloth and Wallaby. Figure S22 (Gorilla and Gibbon) and S23 (Gorilla and Cavia) shows reduced orthology with increase in divergence time. Figure S24 shows relationship between all HGT members in 22 genomes. (PDF 4 MB)

Additional file 2: Table S2: The excel file shows the genomic coordinates of the KRTAP gene repertoires in 22 mammalian species studied. The Gene ID corresponds to the genomic location. (XLS 338 KB)

Additional file 3: Table S3: Gene pairs under significant gene conversion, as detected by GeneConv program. (TXT 49 KB)

Additional file 4: Table S4: Results of RDP3 showing unique recombination events with statistical significance P value of less than 0.01 employing Bonferroni correction. (XLSX 11 KB)
