# Supplementary material for: Mammalian keratin associated proteins (KRTAPs) subgenomes: disentangling hair diversity and adaptation to terrestrial and aquatic environments
Source: BMC Genomics. 2014 Sep 10;15(1):779. doi: 10.1186/1471-2164-15-779 (PMC4180150; doi:10.1186/1471-2164-15-779)

Figure 1

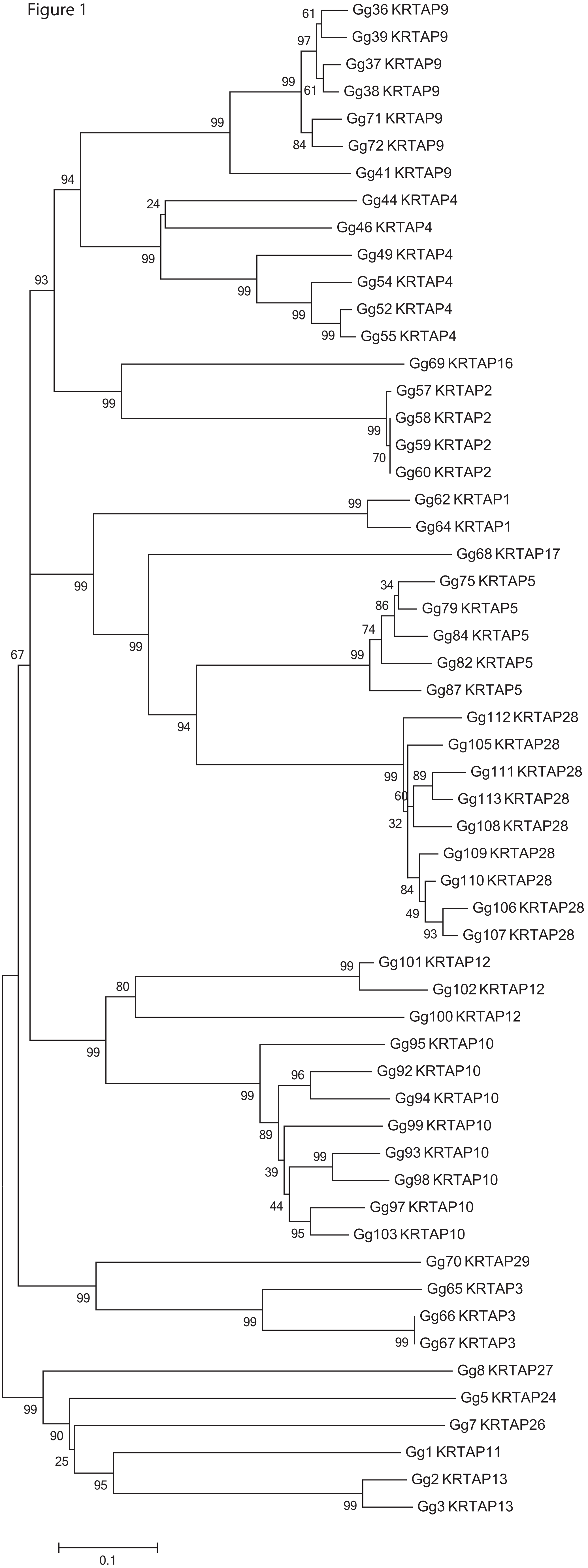

Gg36 KRTAP9
Gg39 KRTAP9
Gg37 KRTAP9
Gg38 KRTAP9
Gg71 KRTAP9
Gg72 KRTAP9
Gg41 KRTAP9
Gg44 KRTAP4
Gg46 KRTAP4
Gg49 KRTAP4
Gg54 KRTAP4
Gg52 KRTAP4
Gg55 KRTAP4
Gg69 KRTAP16
Gg57 KRTAP2
Gg58 KRTAP2
Gg59 KRTAP2
Gg60 KRTAP2
Gg62 KRTAP1
Gg64 KRTAP1
Gg68 KRTAP17
Gg75 KRTAP5
Gg79 KRTAP5
Gg84 KRTAP5
Gg82 KRTAP5
Gg87 KRTAP5
Gg112 KRTAP28
Gg105 KRTAP28
Gg111 KRTAP28
Gg113 KRTAP28
Gg108 KRTAP28
Gg109 KRTAP28
Gg110 KRTAP28
Gg106 KRTAP28
Gg107 KRTAP28
Gg101 KRTAP12
Gg102 KRTAP12
Gg100 KRTAP12
Gg95 KRTAP10
Gg92 KRTAP10
Gg94 KRTAP10
Gg99 KRTAP10
Gg93 KRTAP10
Gg98 KRTAP10
Gg97 KRTAP10
Gg103 KRTAP10
Gg70 KRTAP29
Gg65 KRTAP3
Gg66 KRTAP3
Gg67 KRTAP3
Gg8 KRTAP27
Gg5 KRTAP24
Gg7 KRTAP26
Gg1 KRTAP11
Gg2 KRTAP13
Gg3 KRTAP13
0.1

Figure 2

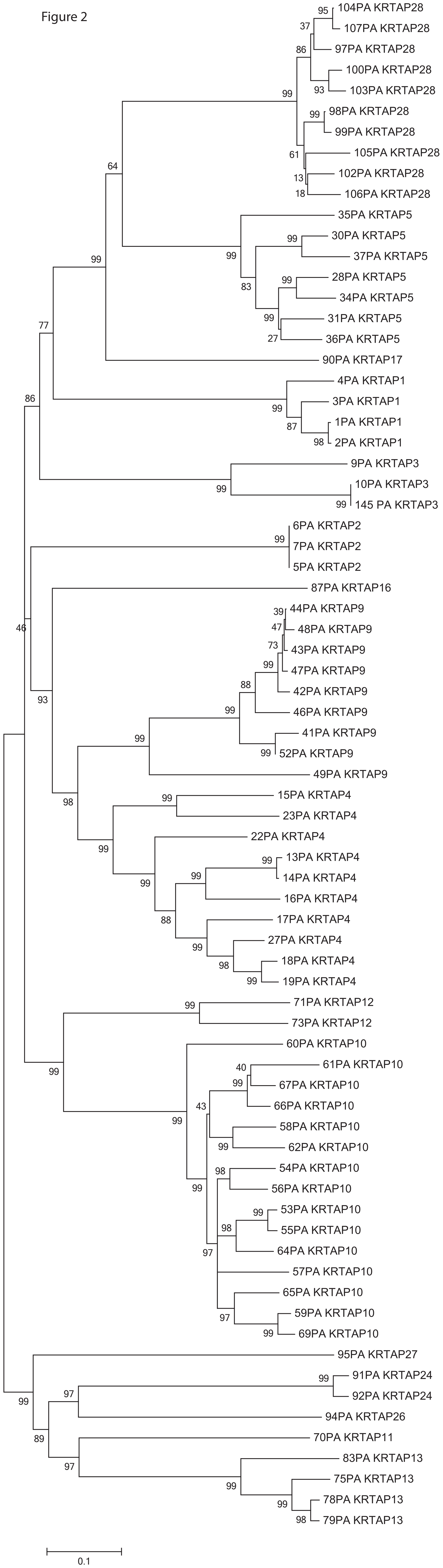
104PA KRTAP28
107PA KRTAP28
97PA KRTAP28
100PA KRTAP28
103PA KRTAP28
98PA KRTAP28
99PA KRTAP28
105PA KRTAP28
102PA KRTAP28
106PA KRTAP28
35PA KRTAP5
30PA KRTAP5
37PA KRTAP5
28PA KRTAP5
34PA KRTAP5
31PA KRTAP5
36PA KRTAP5
90PA KRTAP17
4PA KRTAP1
3PA KRTAP1
1PA KRTAP1
2PA KRTAP1
9PA KRTAP3
10PA KRTAP3
145 PA KRTAP3
6PA KRTAP2
7PA KRTAP2
5PA KRTAP2
87PA KRTAP16
44PA KRTAP9
48PA KRTAP9
43PA KRTAP9
47PA KRTAP9
42PA KRTAP9
46PA KRTAP9
41PA KRTAP9
52PA KRTAP9
49PA KRTAP9
15PA KRTAP4
23PA KRTAP4
22PA KRTAP4
13PA KRTAP4
14PA KRTAP4
16PA KRTAP4
17PA KRTAP4
27PA KRTAP4
18PA KRTAP4
19PA KRTAP4
71PA KRTAP12
73PA KRTAP12
60PA KRTAP10
61PA KRTAP10
67PA KRTAP10
66PA KRTAP10
58PA KRTAP10
62PA KRTAP10
54PA KRTAP10
56PA KRTAP10
53PA KRTAP10
55PA KRTAP10
64PA KRTAP10
57PA KRTAP10
65PA KRTAP10
59PA KRTAP10
69PA KRTAP10
95PA KRTAP27
91PA KRTAP24
92PA KRTAP24
94PA KRTAP26
70PA KRTAP11
83PA KRTAP13
75PA KRTAP13
78PA KRTAP13
79PA KRTAP13
0.1

Figure 3

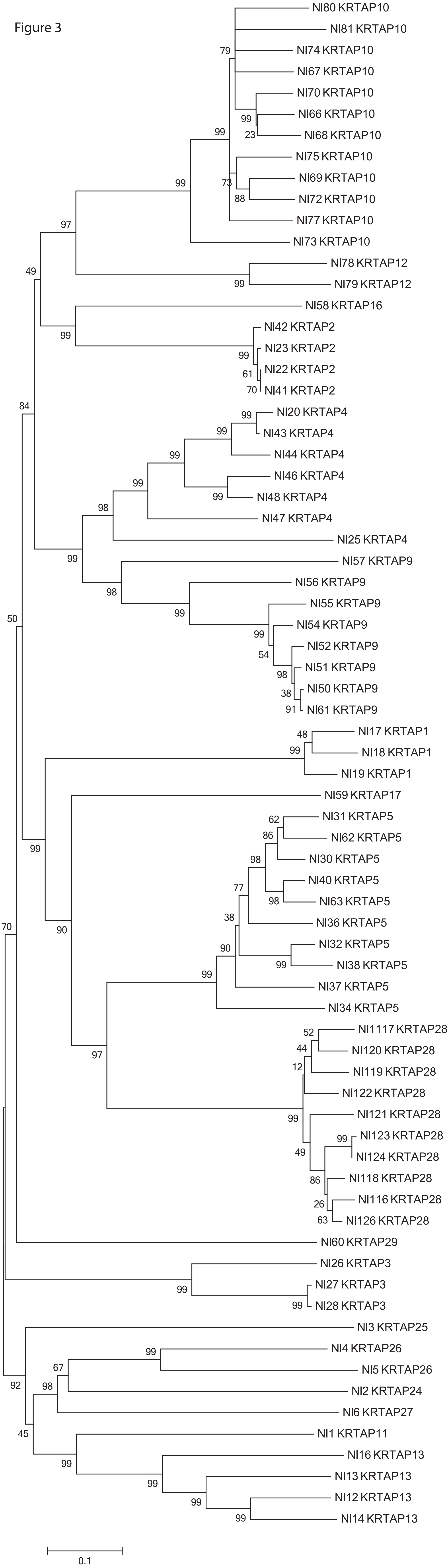
NI80 KRTAP10
NI81 KRTAP10
NI74 KRTAP10
NI67 KRTAP10
NI70 KRTAP10
NI66 KRTAP10
NI68 KRTAP10
NI75 KRTAP10
NI69 KRTAP10
NI72 KRTAP10
NI77 KRTAP10
NI73 KRTAP10
NI78 KRTAP12
NI79 KRTAP12
NI58 KRTAP16
NI42 KRTAP2
NI23 KRTAP2
NI22 KRTAP2
NI41 KRTAP2
NI20 KRTAP4
NI43 KRTAP4
NI44 KRTAP4
NI46 KRTAP4
NI48 KRTAP4
NI47 KRTAP4
NI25 KRTAP4
NI57 KRTAP9
NI56 KRTAP9
NI55 KRTAP9
NI54 KRTAP9
NI52 KRTAP9
NI51 KRTAP9
NI50 KRTAP9
NI61 KRTAP9
NI17 KRTAP1
NI18 KRTAP1
NI19 KRTAP1
NI59 KRTAP17
NI31 KRTAP5
NI62 KRTAP5
NI30 KRTAP5
NI40 KRTAP5
NI63 KRTAP5
NI36 KRTAP5
NI32 KRTAP5
NI38 KRTAP5
NI37 KRTAP5
NI34 KRTAP5
NI1117 KRTAP28
NI120 KRTAP28
NI119 KRTAP28
NI122 KRTAP28
NI121 KRTAP28
NI123 KRTAP28
NI124 KRTAP28
NI118 KRTAP28
NI116 KRTAP28
NI126 KRTAP28
NI60 KRTAP29
NI26 KRTAP3
NI27 KRTAP3
NI28 KRTAP3
NI3 KRTAP25
NI4 KRTAP26
NI5 KRTAP26
NI2 KRTAP24
NI6 KRTAP27
NI1 KRTAP11
NI16 KRTAP13
NI13 KRTAP13
NI12 KRTAP13
NI14 KRTAP13
0.1

Figure 4

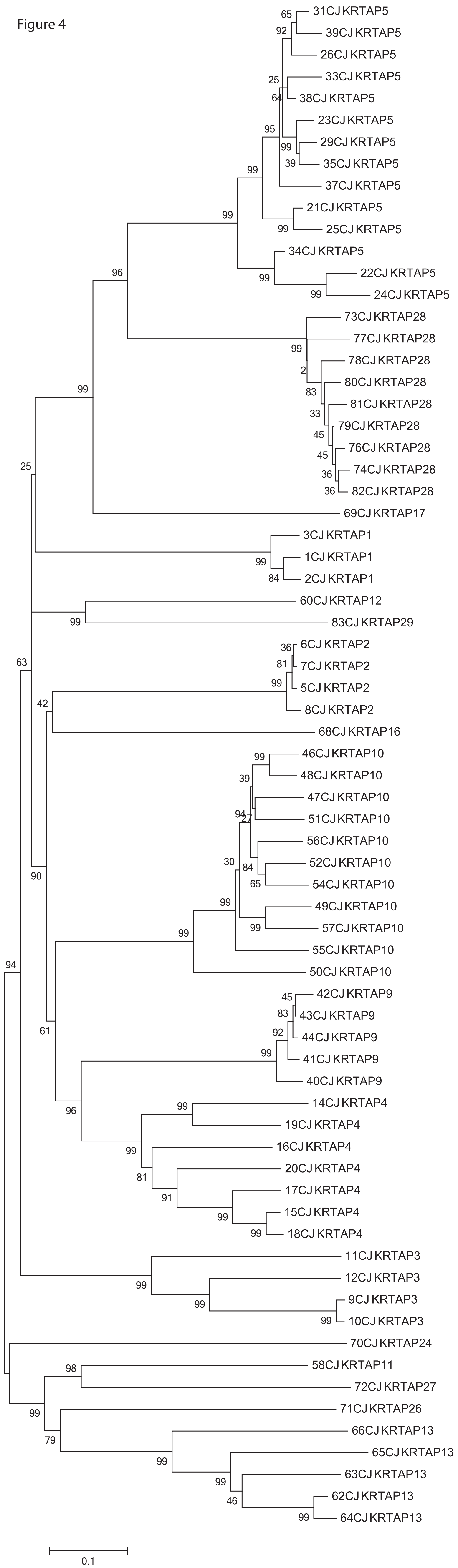
31CJ KRTAP5
39CJ KRTAP5
26CJ KRTAP5
33CJ KRTAP5
38CJ KRTAP5
23CJ KRTAP5
29CJ KRTAP5
35CJ KRTAP5
37CJ KRTAP5
21CJ KRTAP5
25CJ KRTAP5
34CJ KRTAP5
22CJ KRTAP5
24CJ KRTAP5
73CJ KRTAP28
77CJ KRTAP28
78CJ KRTAP28
80CJ KRTAP28
81CJ KRTAP28
79CJ KRTAP28
76CJ KRTAP28
74CJ KRTAP28
82CJ KRTAP28
69CJ KRTAP17
3CJ KRTAP1
1CJ KRTAP1
2CJ KRTAP1
60CJ KRTAP12
83CJ KRTAP29
6CJ KRTAP2
7CJ KRTAP2
5CJ KRTAP2
8CJ KRTAP2
68CJ KRTAP16
46CJ KRTAP10
48CJ KRTAP10
47CJ KRTAP10
51CJ KRTAP10
56CJ KRTAP10
52CJ KRTAP10
54CJ KRTAP10
49CJ KRTAP10
57CJ KRTAP10
55CJ KRTAP10
50CJ KRTAP10
42CJ KRTAP9
43CJ KRTAP9
44CJ KRTAP9
41CJ KRTAP9
40CJ KRTAP9
14CJ KRTAP4
19CJ KRTAP4
16CJ KRTAP4
20CJ KRTAP4
17CJ KRTAP4
15CJ KRTAP4
18CJ KRTAP4
11CJ KRTAP3
12CJ KRTAP3
9CJ KRTAP3
10CJ KRTAP3
70CJ KRTAP24
58CJ KRTAP11
72CJ KRTAP27
71CJ KRTAP26
66CJ KRTAP13
65CJ KRTAP13
63CJ KRTAP13
62CJ KRTAP13
64CJ KRTAP13
0.1

Figure 5

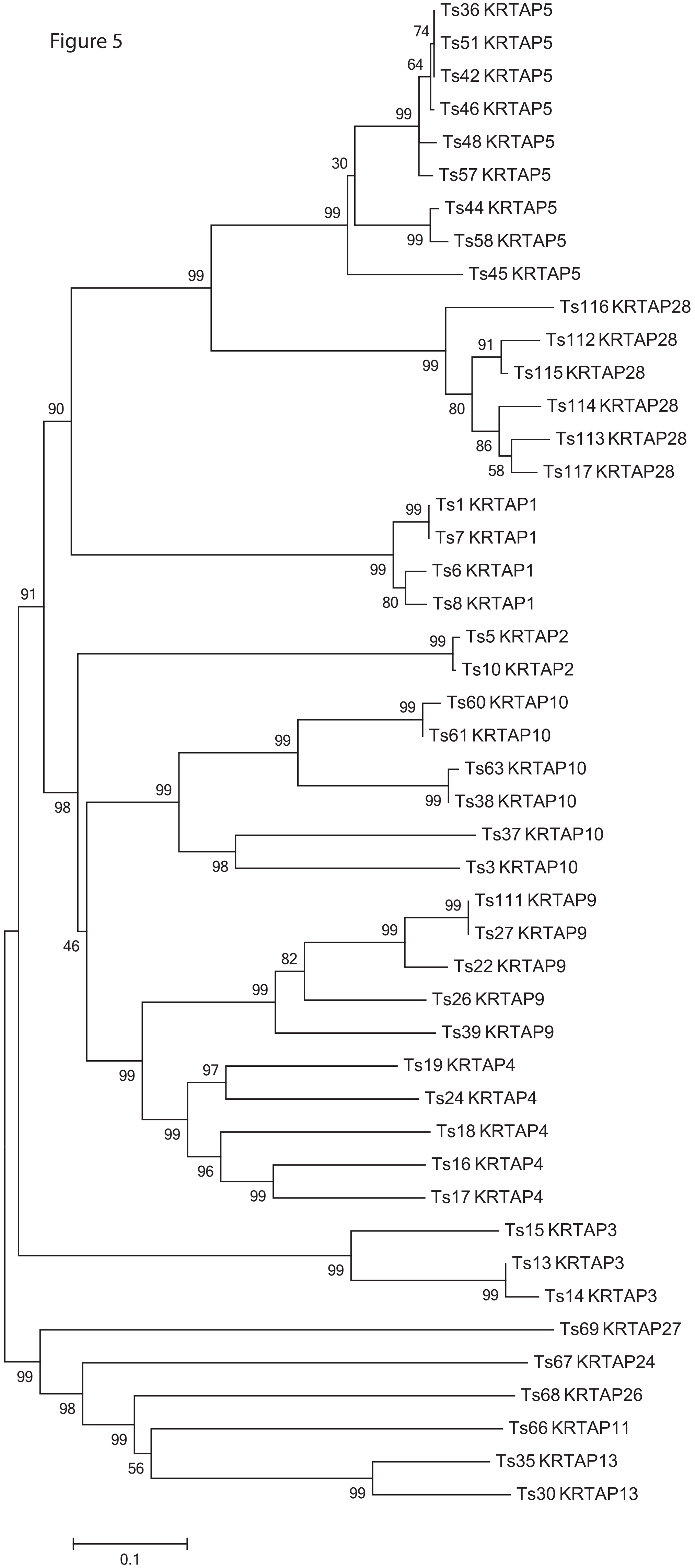
Ts36 KRTAP5
Ts51 KRTAP5
Ts42 KRTAP5
Ts46 KRTAP5
Ts48 KRTAP5
Ts57 KRTAP5
Ts44 KRTAP5
Ts58 KRTAP5
Ts45 KRTAP5
Ts116 KRTAP28
Ts112 KRTAP28
Ts115 KRTAP28
Ts114 KRTAP28
Ts113 KRTAP28
Ts117 KRTAP28
Ts1 KRTAP1
Ts7 KRTAP1
Ts6 KRTAP1
Ts8 KRTAP1
Ts5 KRTAP2
Ts10 KRTAP2
Ts60 KRTAP10
Ts61 KRTAP10
Ts63 KRTAP10
Ts38 KRTAP10
Ts37 KRTAP10
Ts3 KRTAP10
Ts111 KRTAP9
Ts27 KRTAP9
Ts22 KRTAP9
Ts26 KRTAP9
Ts39 KRTAP9
Ts19 KRTAP4
Ts24 KRTAP4
Ts18 KRTAP4
Ts16 KRTAP4
Ts17 KRTAP4
Ts15 KRTAP3
Ts13 KRTAP3
Ts14 KRTAP3
Ts69 KRTAP27
Ts67 KRTAP24
Ts68 KRTAP26
Ts66 KRTAP11
Ts35 KRTAP13
Ts30 KRTAP13
0.1

Figure 6

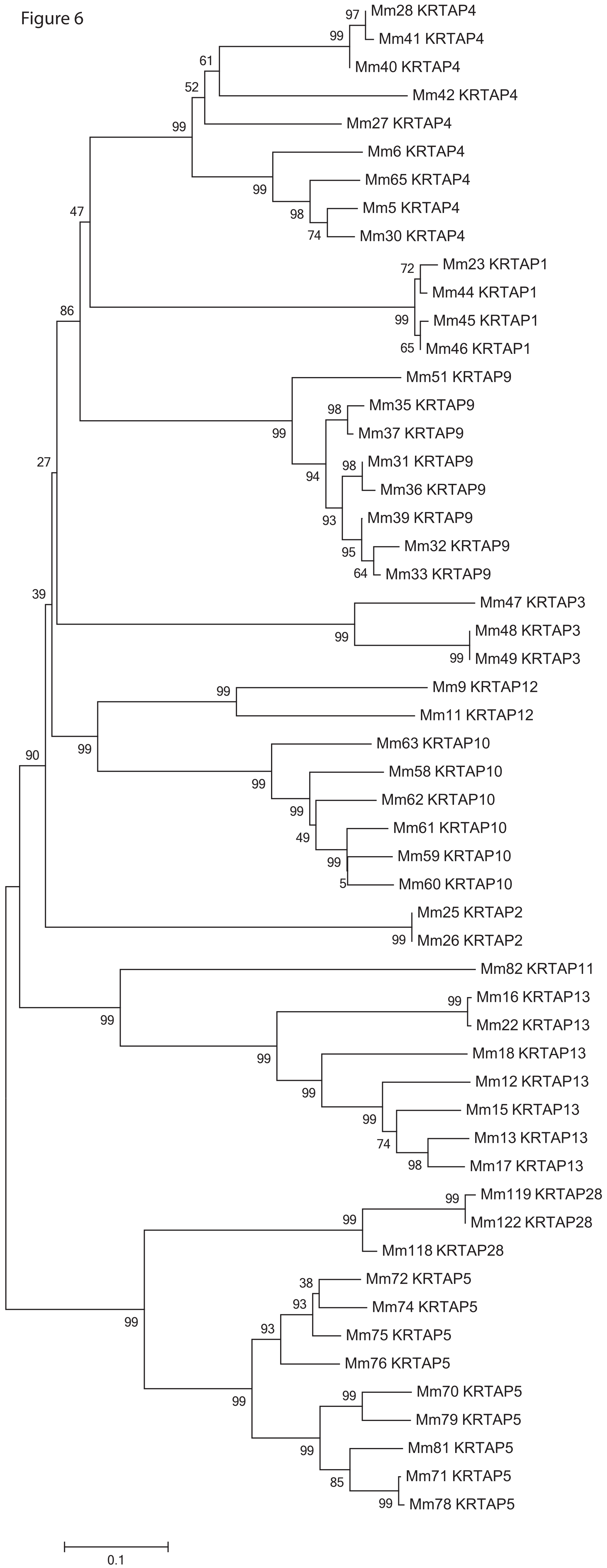

Mm28 KRTAP4
Mm41 KRTAP4
Mm40 KRTAP4
Mm42 KRTAP4
Mm27 KRTAP4
Mm6 KRTAP4
Mm65 KRTAP4
Mm5 KRTAP4
Mm30 KRTAP4
Mm23 KRTAP1
Mm44 KRTAP1
Mm45 KRTAP1
Mm46 KRTAP1
Mm51 KRTAP9
Mm35 KRTAP9
Mm37 KRTAP9
Mm31 KRTAP9
Mm36 KRTAP9
Mm39 KRTAP9
Mm32 KRTAP9
Mm33 KRTAP9
Mm47 KRTAP3
Mm48 KRTAP3
Mm49 KRTAP3
Mm9 KRTAP12
Mm11 KRTAP12
Mm63 KRTAP10
Mm58 KRTAP10
Mm62 KRTAP10
Mm61 KRTAP10
Mm59 KRTAP10
Mm60 KRTAP10
Mm25 KRTAP2
Mm26 KRTAP2
Mm82 KRTAP11
Mm16 KRTAP13
Mm22 KRTAP13
Mm18 KRTAP13
Mm12 KRTAP13
Mm15 KRTAP13
Mm13 KRTAP13
Mm17 KRTAP13
Mm119 KRTAP28
Mm122 KRTAP28
Mm118 KRTAP28
Mm72 KRTAP5
Mm74 KRTAP5
Mm75 KRTAP5
Mm76 KRTAP5
Mm70 KRTAP5
Mm79 KRTAP5
Mm81 KRTAP5
Mm71 KRTAP5
Mm78 KRTAP5
0.1

Figure 7

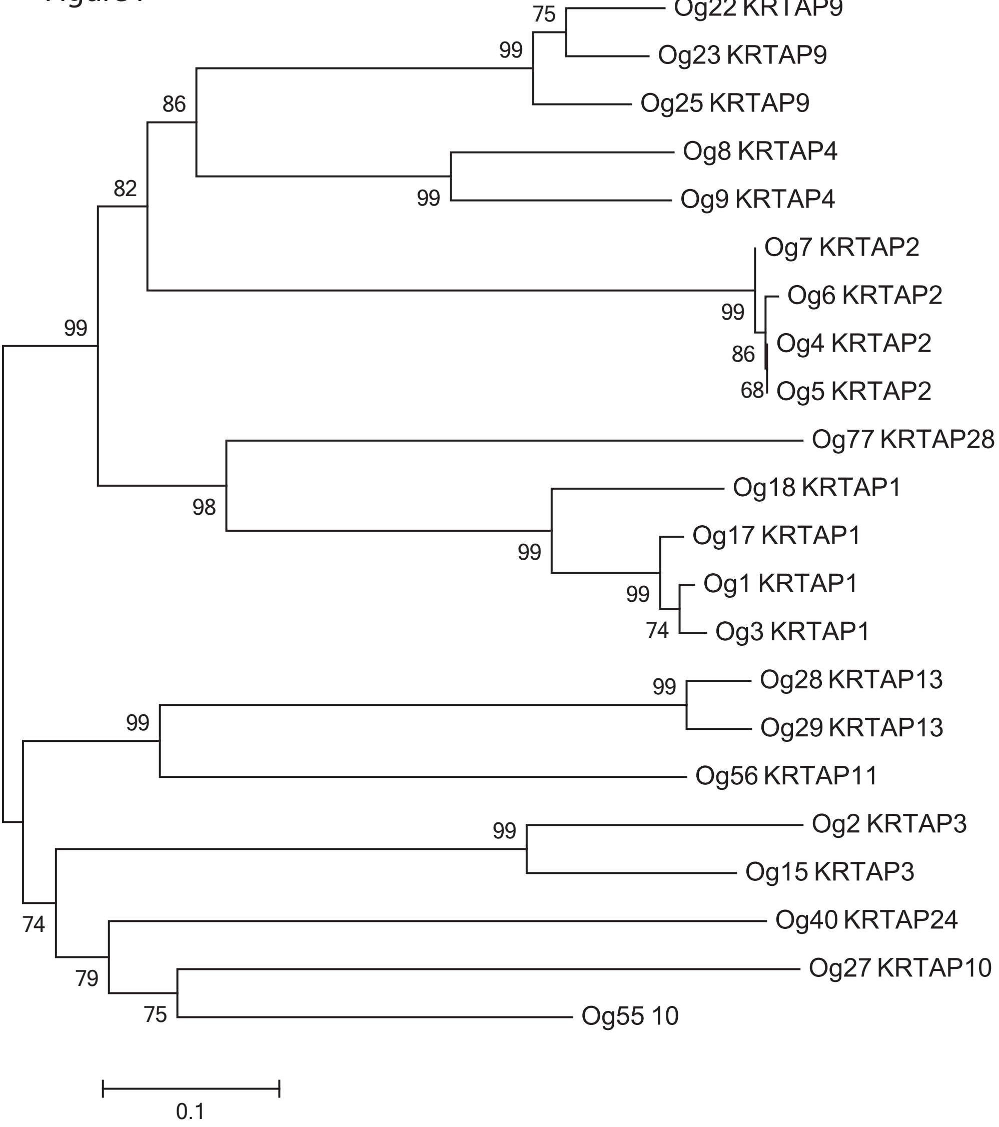
Og22 KRTAP9
Og23 KRTAP9
Og25 KRTAP9
Og8 KRTAP4
Og9 KRTAP4
Og7 KRTAP2
Og6 KRTAP2
Og4 KRTAP2
Og5 KRTAP2
Og77 KRTAP28
Og18 KRTAP1
Og17 KRTAP1
Og1 KRTAP1
Og3 KRTAP1
Og28 KRTAP13
Og29 KRTAP13
Og56 KRTAP11
Og2 KRTAP3
Og15 KRTAP3
Og40 KRTAP24
Og27 KRTAP10
Og55 10
75
99
86
99
82
99
99
86
68
98
99
99
74
99
99
99
74
79
75
0.1

Figure 8

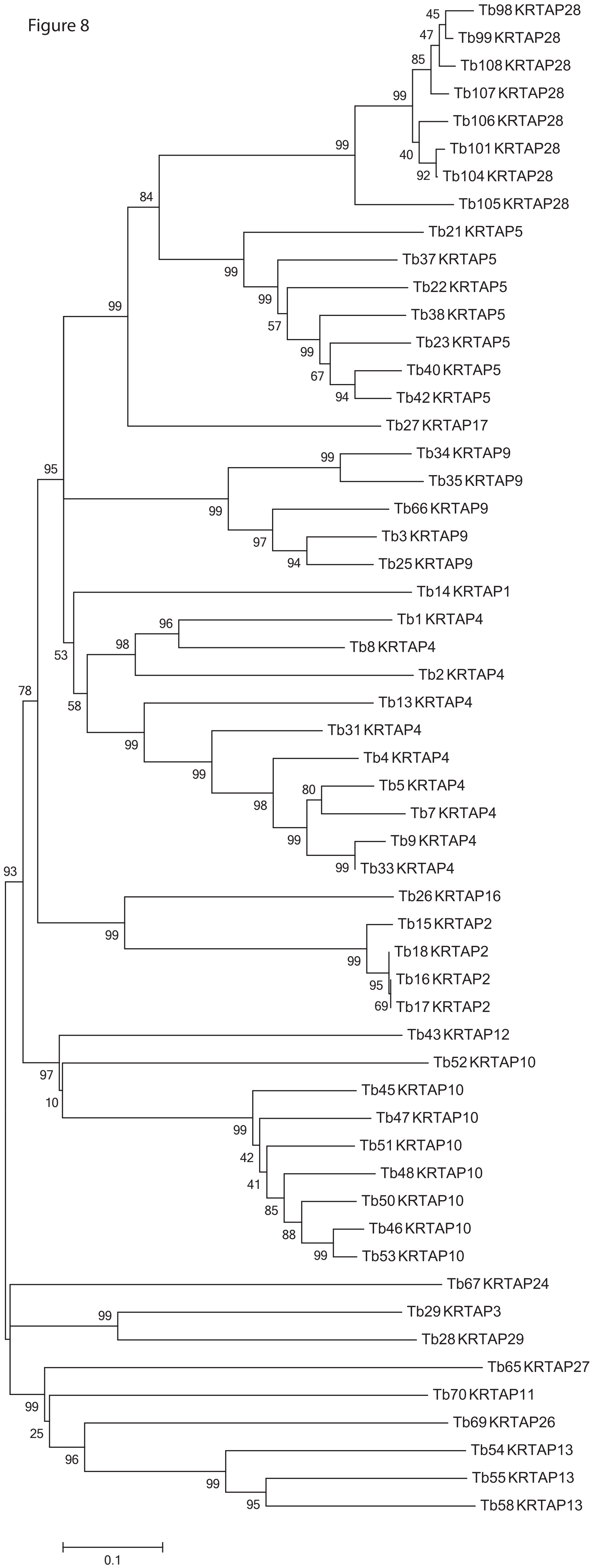

Tb98 KRTAP28
Tb99 KRTAP28
Tb108 KRTAP28
Tb107 KRTAP28
Tb106 KRTAP28
Tb101 KRTAP28
Tb104 KRTAP28
Tb105 KRTAP28
Tb21 KRTAP5
Tb37 KRTAP5
Tb22 KRTAP5
Tb38 KRTAP5
Tb23 KRTAP5
Tb40 KRTAP5
Tb42 KRTAP5
Tb27 KRTAP17
Tb34 KRTAP9
Tb35 KRTAP9
Tb66 KRTAP9
Tb3 KRTAP9
Tb25 KRTAP9
Tb14 KRTAP1
Tb1 KRTAP4
Tb8 KRTAP4
Tb2 KRTAP4
Tb13 KRTAP4
Tb31 KRTAP4
Tb4 KRTAP4
Tb5 KRTAP4
Tb7 KRTAP4
Tb9 KRTAP4
Tb33 KRTAP4
Tb26 KRTAP16
Tb15 KRTAP2
Tb18 KRTAP2
Tb16 KRTAP2
Tb17 KRTAP2
Tb43 KRTAP12
Tb52 KRTAP10
Tb45 KRTAP10
Tb47 KRTAP10
Tb51 KRTAP10
Tb48 KRTAP10
Tb50 KRTAP10
Tb46 KRTAP10
Tb53 KRTAP10
Tb67 KRTAP24
Tb29 KRTAP3
Tb28 KRTAP29
Tb65 KRTAP27
Tb70 KRTAP11
Tb69 KRTAP26
Tb54 KRTAP13
Tb55 KRTAP13
Tb58 KRTAP13
0.1

Figure 9

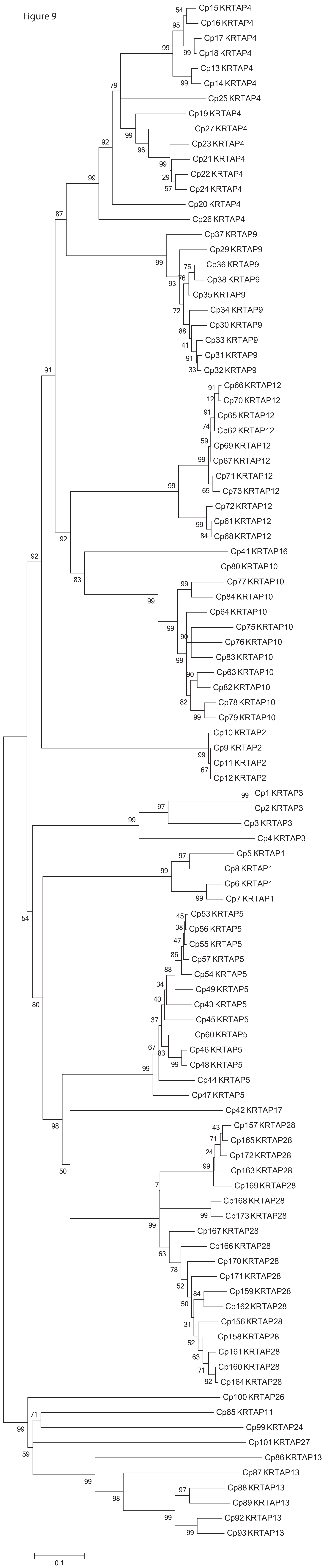
Cp15 KRTAP4
Cp16 KRTAP4
Cp17 KRTAP4
Cp18 KRTAP4
Cp13 KRTAP4
Cp14 KRTAP4
Cp25 KRTAP4
Cp19 KRTAP4
Cp27 KRTAP4
Cp23 KRTAP4
Cp21 KRTAP4
Cp22 KRTAP4
Cp24 KRTAP4
Cp20 KRTAP4
Cp26 KRTAP4
Cp37 KRTAP9
Cp29 KRTAP9
Cp36 KRTAP9
Cp38 KRTAP9
Cp35 KRTAP9
Cp34 KRTAP9
Cp30 KRTAP9
Cp33 KRTAP9
Cp31 KRTAP9
Cp32 KRTAP9
Cp66 KRTAP12
Cp70 KRTAP12
Cp65 KRTAP12
Cp62 KRTAP12
Cp69 KRTAP12
Cp67 KRTAP12
Cp71 KRTAP12
Cp73 KRTAP12
Cp72 KRTAP12
Cp61 KRTAP12
Cp68 KRTAP12
Cp41 KRTAP16
Cp80 KRTAP10
Cp77 KRTAP10
Cp84 KRTAP10
Cp64 KRTAP10
Cp75 KRTAP10
Cp76 KRTAP10
Cp83 KRTAP10
Cp63 KRTAP10
Cp82 KRTAP10
Cp78 KRTAP10
Cp79 KRTAP10
Cp10 KRTAP2
Cp9 KRTAP2
Cp11 KRTAP2
Cp12 KRTAP2
Cp1 KRTAP3
Cp2 KRTAP3
Cp3 KRTAP3
Cp4 KRTAP3
Cp5 KRTAP1
Cp8 KRTAP1
Cp6 KRTAP1
Cp7 KRTAP1
Cp53 KRTAP5
Cp56 KRTAP5
Cp55 KRTAP5
Cp57 KRTAP5
Cp54 KRTAP5
Cp49 KRTAP5
Cp43 KRTAP5
Cp45 KRTAP5
Cp60 KRTAP5
Cp46 KRTAP5
Cp48 KRTAP5
Cp44 KRTAP5
Cp47 KRTAP5
Cp42 KRTAP17
Cp157 KRTAP28
Cp165 KRTAP28
Cp172 KRTAP28
Cp163 KRTAP28
Cp169 KRTAP28
Cp168 KRTAP28
Cp173 KRTAP28
Cp167 KRTAP28
Cp166 KRTAP28
Cp170 KRTAP28
Cp171 KRTAP28
Cp159 KRTAP28
Cp162 KRTAP28
Cp156 KRTAP28
Cp158 KRTAP28
Cp161 KRTAP28
Cp160 KRTAP28
Cp164 KRTAP28
Cp100 KRTAP26
Cp85 KRTAP11
Cp99 KRTAP24
Cp101 KRTAP27
Cp86 KRTAP13
Cp87 KRTAP13
Cp88 KRTAP13
Cp89 KRTAP13
Cp92 KRTAP13
Cp93 KRTAP13
0.1

Figure 10

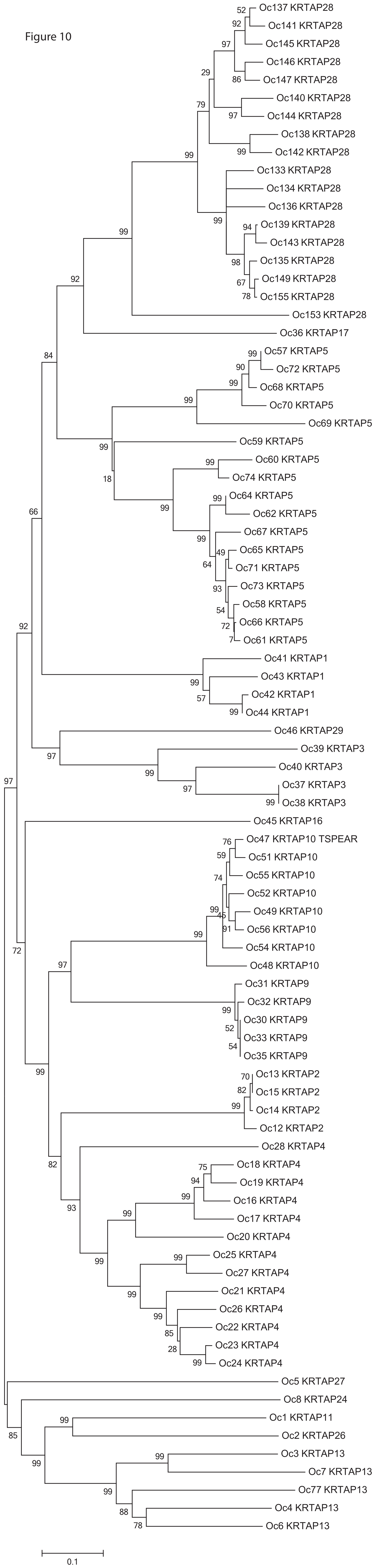

Oc137 KRTAP28
Oc141 KRTAP28
Oc145 KRTAP28
Oc146 KRTAP28
Oc147 KRTAP28
Oc140 KRTAP28
Oc144 KRTAP28
Oc138 KRTAP28
Oc142 KRTAP28
Oc133 KRTAP28
Oc134 KRTAP28
Oc136 KRTAP28
Oc139 KRTAP28
Oc143 KRTAP28
Oc135 KRTAP28
Oc149 KRTAP28
Oc155 KRTAP28
Oc153 KRTAP28
Oc36 KRTAP17
Oc57 KRTAP5
Oc72 KRTAP5
Oc68 KRTAP5
Oc70 KRTAP5
Oc69 KRTAP5
Oc59 KRTAP5
Oc60 KRTAP5
Oc74 KRTAP5
Oc64 KRTAP5
Oc62 KRTAP5
Oc67 KRTAP5
Oc65 KRTAP5
Oc71 KRTAP5
Oc73 KRTAP5
Oc58 KRTAP5
Oc66 KRTAP5
Oc61 KRTAP5
Oc41 KRTAP1
Oc43 KRTAP1
Oc42 KRTAP1
Oc44 KRTAP1
Oc46 KRTAP29
Oc39 KRTAP3
Oc40 KRTAP3
Oc37 KRTAP3
Oc38 KRTAP3
Oc45 KRTAP16
Oc47 KRTAP10 TSPEAR
Oc51 KRTAP10
Oc55 KRTAP10
Oc52 KRTAP10
Oc49 KRTAP10
Oc56 KRTAP10
Oc54 KRTAP10
Oc48 KRTAP10
Oc31 KRTAP9
Oc32 KRTAP9
Oc30 KRTAP9
Oc33 KRTAP9
Oc35 KRTAP9
Oc13 KRTAP2
Oc15 KRTAP2
Oc14 KRTAP2
Oc12 KRTAP2
Oc28 KRTAP4
Oc18 KRTAP4
Oc19 KRTAP4
Oc16 KRTAP4
Oc17 KRTAP4
Oc20 KRTAP4
Oc25 KRTAP4
Oc27 KRTAP4
Oc21 KRTAP4
Oc26 KRTAP4
Oc22 KRTAP4
Oc23 KRTAP4
Oc24 KRTAP4
Oc5 KRTAP27
Oc8 KRTAP24
Oc1 KRTAP11
Oc2 KRTAP26
Oc3 KRTAP13
Oc7 KRTAP13
Oc77 KRTAP13
Oc4 KRTAP13
Oc6 KRTAP13
0.1

Figure 11

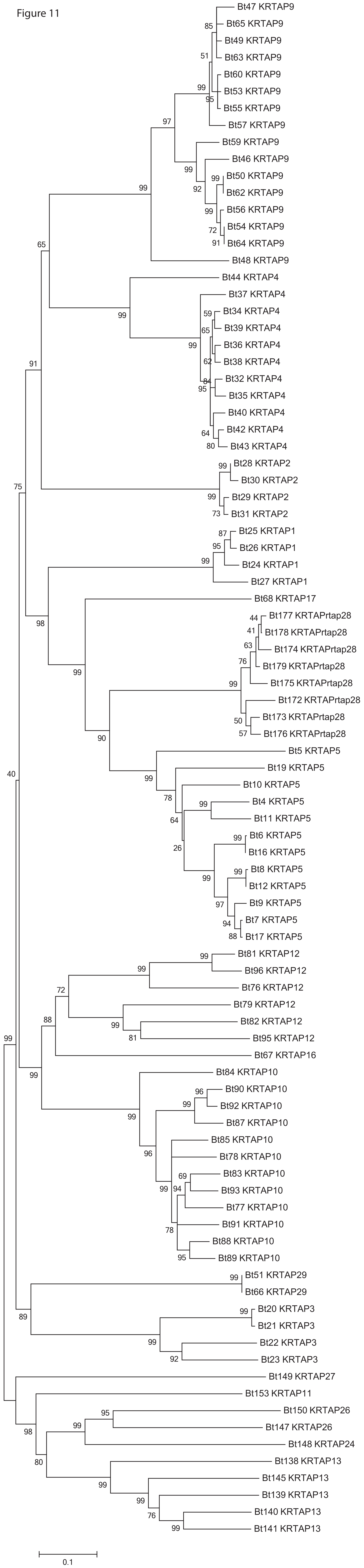
Bt47 KRTAP9
Bt65 KRTAP9
Bt49 KRTAP9
Bt63 KRTAP9
Bt60 KRTAP9
Bt53 KRTAP9
Bt55 KRTAP9
Bt57 KRTAP9
Bt59 KRTAP9
Bt46 KRTAP9
Bt50 KRTAP9
Bt62 KRTAP9
Bt56 KRTAP9
Bt54 KRTAP9
Bt64 KRTAP9
Bt48 KRTAP9
Bt44 KRTAP4
Bt37 KRTAP4
Bt34 KRTAP4
Bt39 KRTAP4
Bt36 KRTAP4
Bt38 KRTAP4
Bt32 KRTAP4
Bt35 KRTAP4
Bt40 KRTAP4
Bt42 KRTAP4
Bt43 KRTAP4
Bt28 KRTAP2
Bt30 KRTAP2
Bt29 KRTAP2
Bt31 KRTAP2
Bt25 KRTAP1
Bt26 KRTAP1
Bt24 KRTAP1
Bt27 KRTAP1
Bt68 KRTAP17
Bt177 KRTAPrtap28
Bt178 KRTAPrtap28
Bt174 KRTAPrtap28
Bt179 KRTAPrtap28
Bt175 KRTAPrtap28
Bt172 KRTAPrtap28
Bt173 KRTAPrtap28
Bt176 KRTAPrtap28
Bt5 KRTAP5
Bt19 KRTAP5
Bt10 KRTAP5
Bt4 KRTAP5
Bt11 KRTAP5
Bt6 KRTAP5
Bt16 KRTAP5
Bt8 KRTAP5
Bt12 KRTAP5
Bt9 KRTAP5
Bt7 KRTAP5
Bt17 KRTAP5
Bt81 KRTAP12
Bt96 KRTAP12
Bt76 KRTAP12
Bt79 KRTAP12
Bt82 KRTAP12
Bt95 KRTAP12
Bt67 KRTAP16
Bt84 KRTAP10
Bt90 KRTAP10
Bt92 KRTAP10
Bt87 KRTAP10
Bt85 KRTAP10
Bt78 KRTAP10
Bt83 KRTAP10
Bt93 KRTAP10
Bt77 KRTAP10
Bt91 KRTAP10
Bt88 KRTAP10
Bt89 KRTAP10
Bt51 KRTAP29
Bt66 KRTAP29
Bt20 KRTAP3
Bt21 KRTAP3
Bt22 KRTAP3
Bt23 KRTAP3
Bt149 KRTAP27
Bt153 KRTAP11
Bt150 KRTAP26
Bt147 KRTAP26
Bt148 KRTAP24
Bt138 KRTAP13
Bt145 KRTAP13
Bt139 KRTAP13
Bt140 KRTAP13
Bt141 KRTAP13
0.1

Figure 12

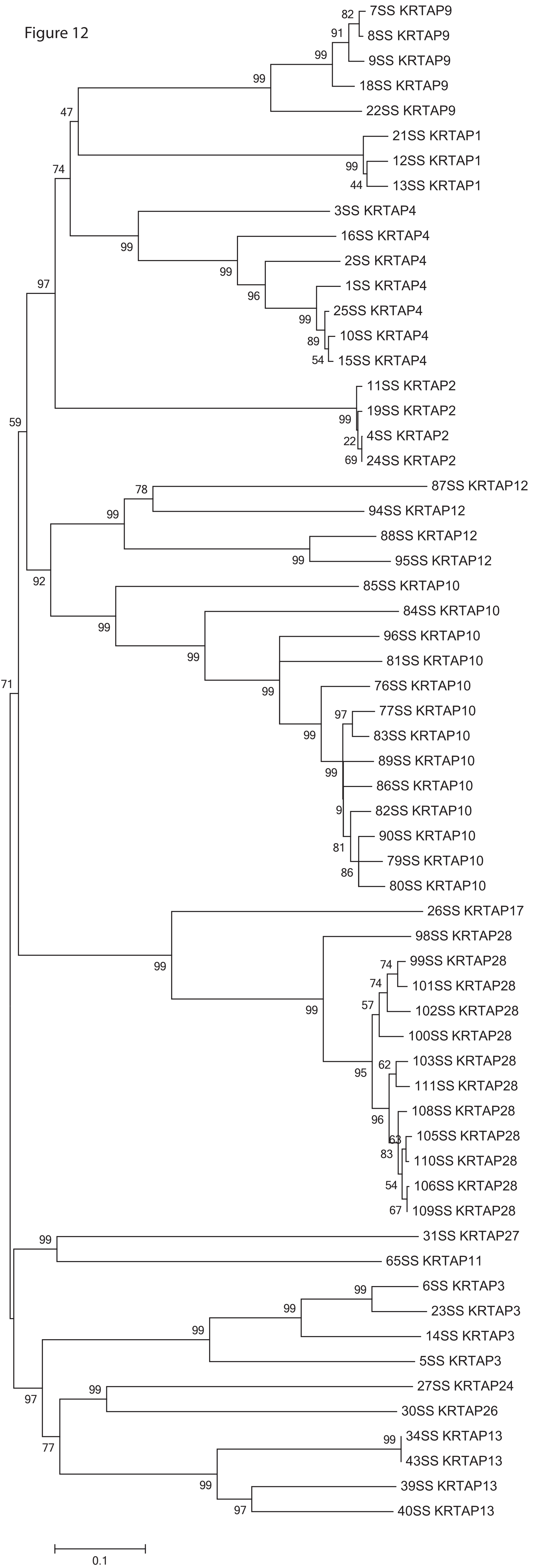
7SS KRTAP9
8SS KRTAP9
9SS KRTAP9
18SS KRTAP9
22SS KRTAP9
21SS KRTAP1
12SS KRTAP1
13SS KRTAP1
3SS KRTAP4
16SS KRTAP4
2SS KRTAP4
1SS KRTAP4
25SS KRTAP4
10SS KRTAP4
15SS KRTAP4
11SS KRTAP2
19SS KRTAP2
4SS KRTAP2
24SS KRTAP2
87SS KRTAP12
94SS KRTAP12
88SS KRTAP12
95SS KRTAP12
85SS KRTAP10
84SS KRTAP10
96SS KRTAP10
81SS KRTAP10
76SS KRTAP10
77SS KRTAP10
83SS KRTAP10
89SS KRTAP10
86SS KRTAP10
82SS KRTAP10
90SS KRTAP10
79SS KRTAP10
80SS KRTAP10
26SS KRTAP17
98SS KRTAP28
99SS KRTAP28
101SS KRTAP28
102SS KRTAP28
100SS KRTAP28
103SS KRTAP28
111SS KRTAP28
108SS KRTAP28
105SS KRTAP28
110SS KRTAP28
106SS KRTAP28
109SS KRTAP28
31SS KRTAP27
65SS KRTAP11
6SS KRTAP3
23SS KRTAP3
14SS KRTAP3
5SS KRTAP3
27SS KRTAP24
30SS KRTAP26
34SS KRTAP13
43SS KRTAP13
39SS KRTAP13
40SS KRTAP13
0.1

Figure 13

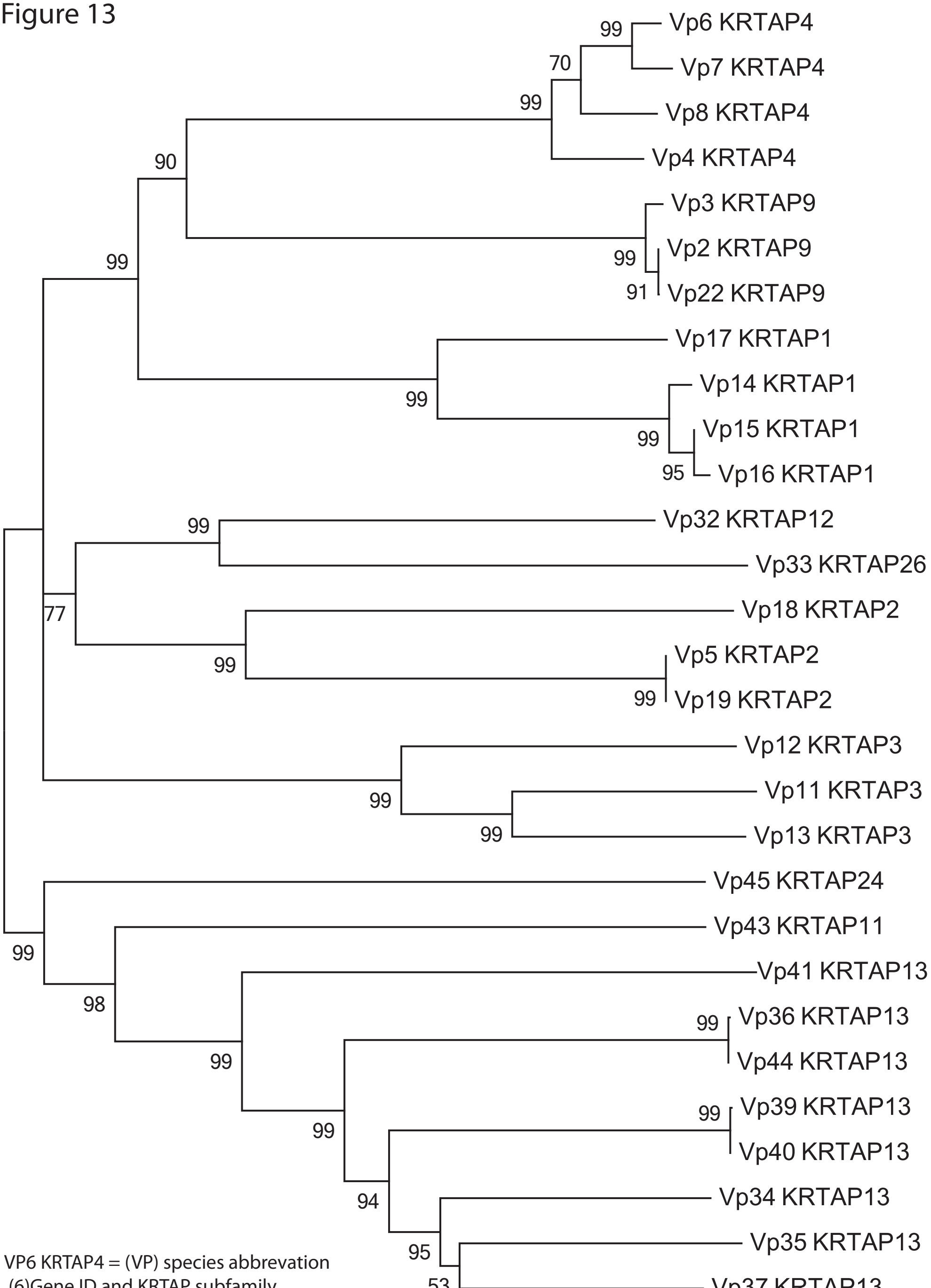

VP6 KRTAP4 = (VP) species abbrevation
(6)Gene ID and KRTAP subfamily

Figure 14

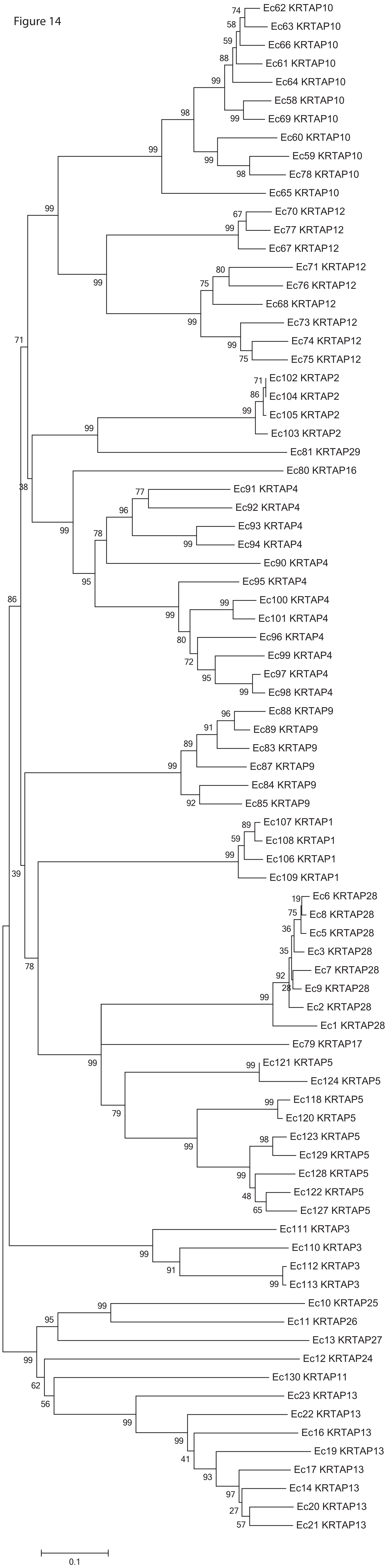
Ec62 KRTAP10
Ec63 KRTAP10
Ec66 KRTAP10
Ec61 KRTAP10
Ec64 KRTAP10
Ec58 KRTAP10
Ec69 KRTAP10
Ec60 KRTAP10
Ec59 KRTAP10
Ec78 KRTAP10
Ec65 KRTAP10
Ec70 KRTAP12
Ec77 KRTAP12
Ec67 KRTAP12
Ec71 KRTAP12
Ec76 KRTAP12
Ec68 KRTAP12
Ec73 KRTAP12
Ec74 KRTAP12
Ec75 KRTAP12
Ec102 KRTAP2
Ec104 KRTAP2
Ec105 KRTAP2
Ec103 KRTAP2
Ec81 KRTAP29
Ec80 KRTAP16
Ec91 KRTAP4
Ec92 KRTAP4
Ec93 KRTAP4
Ec94 KRTAP4
Ec90 KRTAP4
Ec95 KRTAP4
Ec100 KRTAP4
Ec101 KRTAP4
Ec96 KRTAP4
Ec99 KRTAP4
Ec97 KRTAP4
Ec98 KRTAP4
Ec88 KRTAP9
Ec89 KRTAP9
Ec83 KRTAP9
Ec87 KRTAP9
Ec84 KRTAP9
Ec85 KRTAP9
Ec107 KRTAP1
Ec108 KRTAP1
Ec106 KRTAP1
Ec109 KRTAP1
Ec6 KRTAP28
Ec8 KRTAP28
Ec5 KRTAP28
Ec3 KRTAP28
Ec7 KRTAP28
Ec9 KRTAP28
Ec2 KRTAP28
Ec1 KRTAP28
Ec79 KRTAP17
Ec121 KRTAP5
Ec124 KRTAP5
Ec118 KRTAP5
Ec120 KRTAP5
Ec123 KRTAP5
Ec129 KRTAP5
Ec128 KRTAP5
Ec122 KRTAP5
Ec127 KRTAP5
Ec111 KRTAP3
Ec110 KRTAP3
Ec112 KRTAP3
Ec113 KRTAP3
Ec10 KRTAP25
Ec11 KRTAP26
Ec13 KRTAP27
Ec12 KRTAP24
Ec130 KRTAP11
Ec23 KRTAP13
Ec22 KRTAP13
Ec16 KRTAP13
Ec19 KRTAP13
Ec17 KRTAP13
Ec14 KRTAP13
Ec20 KRTAP13
Ec21 KRTAP13
0.1

Figure 15

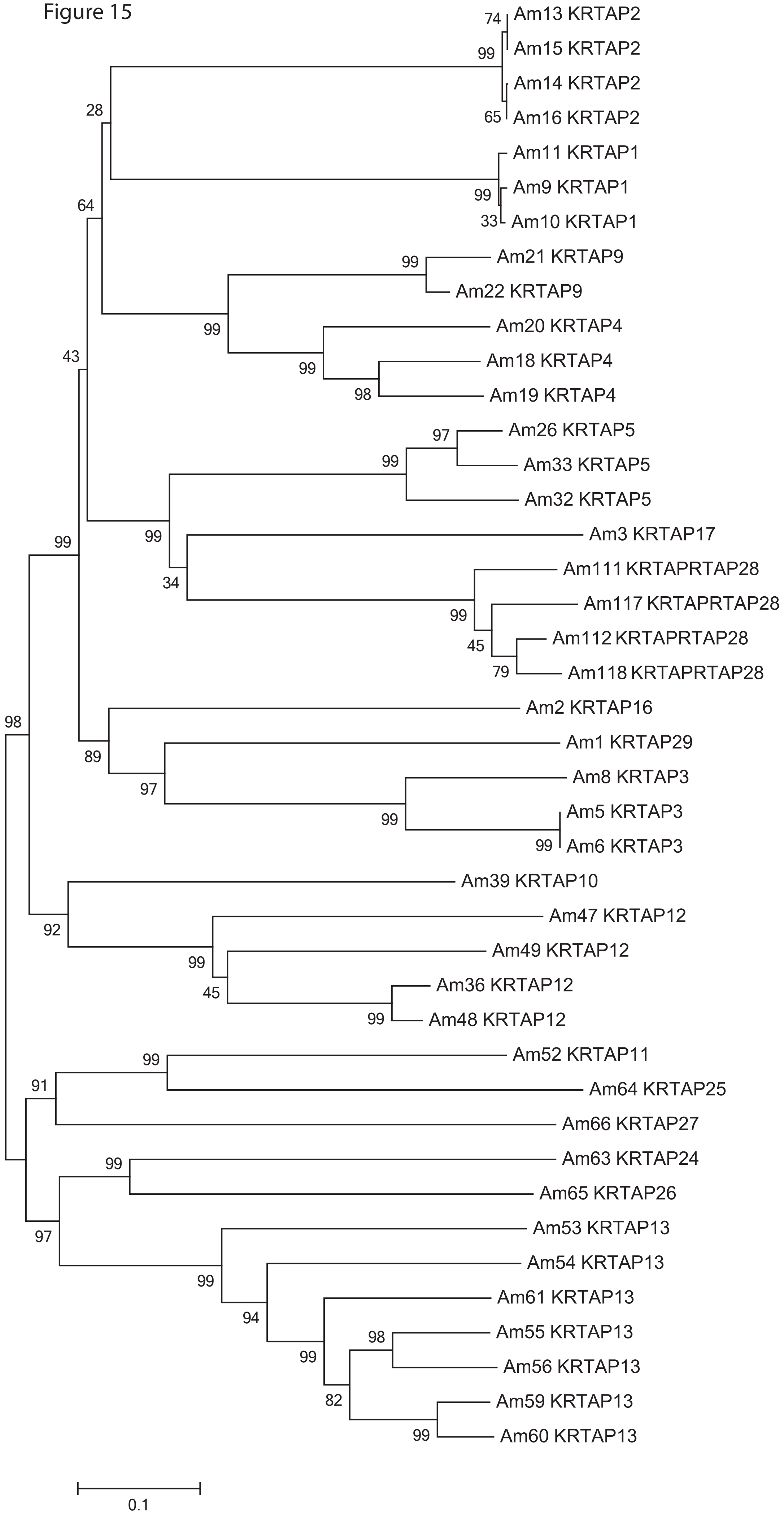

Am13 KRTAP2
Am15 KRTAP2
Am14 KRTAP2
Am16 KRTAP2
Am11 KRTAP1
Am9 KRTAP1
Am10 KRTAP1
Am21 KRTAP9
Am22 KRTAP9
Am20 KRTAP4
Am18 KRTAP4
Am19 KRTAP4
Am26 KRTAP5
Am33 KRTAP5
Am32 KRTAP5
Am3 KRTAP17
Am111 KRTAPRTAP28
Am117 KRTAPRTAP28
Am112 KRTAPRTAP28
Am118 KRTAPRTAP28
Am2 KRTAP16
Am1 KRTAP29
Am8 KRTAP3
Am5 KRTAP3
Am6 KRTAP3
Am39 KRTAP10
Am47 KRTAP12
Am49 KRTAP12
Am36 KRTAP12
Am48 KRTAP12
Am52 KRTAP11
Am64 KRTAP25
Am66 KRTAP27
Am63 KRTAP24
Am65 KRTAP26
Am53 KRTAP13
Am54 KRTAP13
Am61 KRTAP13
Am55 KRTAP13
Am56 KRTAP13
Am59 KRTAP13
Am60 KRTAP13
0.1

Figure 16

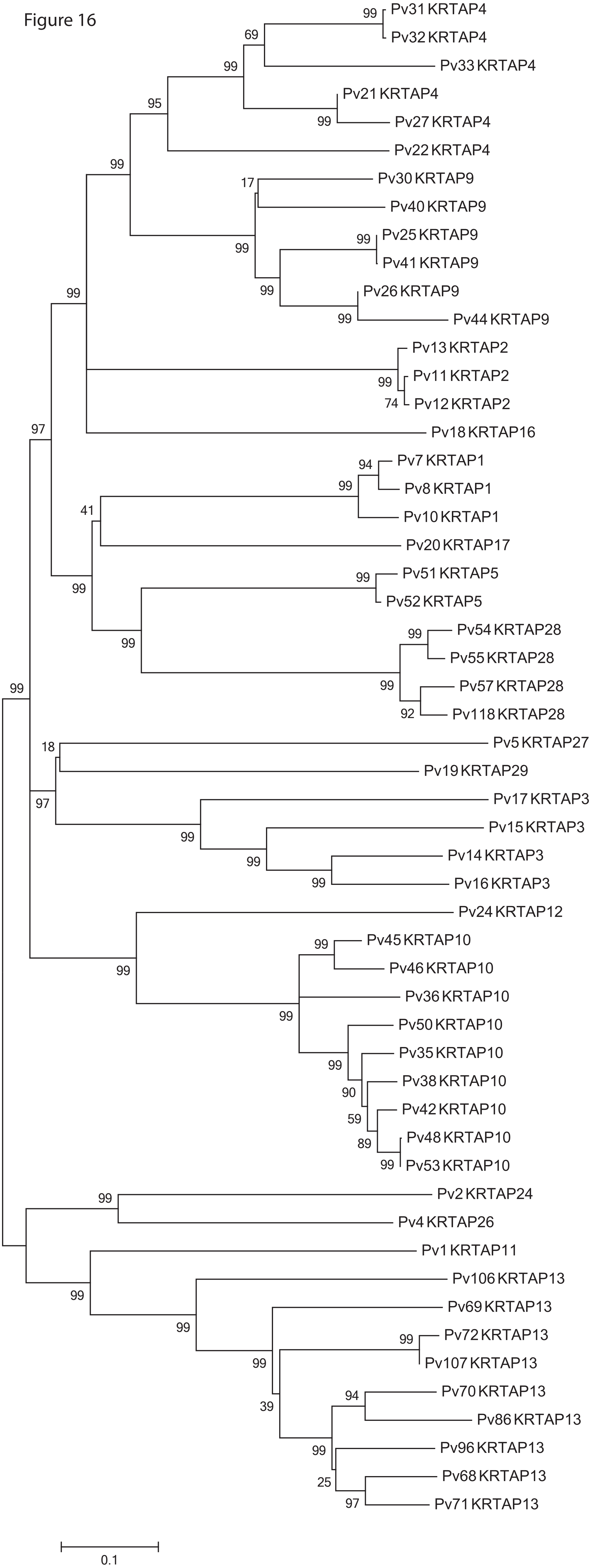
Pv31 KRTAP4
Pv32 KRTAP4
Pv33 KRTAP4
Pv21 KRTAP4
Pv27 KRTAP4
Pv22 KRTAP4
Pv30 KRTAP9
Pv40 KRTAP9
Pv25 KRTAP9
Pv41 KRTAP9
Pv26 KRTAP9
Pv44 KRTAP9
Pv13 KRTAP2
Pv11 KRTAP2
Pv12 KRTAP2
Pv18 KRTAP16
Pv7 KRTAP1
Pv8 KRTAP1
Pv10 KRTAP1
Pv20 KRTAP17
Pv51 KRTAP5
Pv52 KRTAP5
Pv54 KRTAP28
Pv55 KRTAP28
Pv57 KRTAP28
Pv118 KRTAP28
Pv5 KRTAP27
Pv19 KRTAP29
Pv17 KRTAP3
Pv15 KRTAP3
Pv14 KRTAP3
Pv16 KRTAP3
Pv24 KRTAP12
Pv45 KRTAP10
Pv46 KRTAP10
Pv36 KRTAP10
Pv50 KRTAP10
Pv35 KRTAP10
Pv38 KRTAP10
Pv42 KRTAP10
Pv48 KRTAP10
Pv53 KRTAP10
Pv2 KRTAP24
Pv4 KRTAP26
Pv1 KRTAP11
Pv106 KRTAP13
Pv69 KRTAP13
Pv72 KRTAP13
Pv107 KRTAP13
Pv70 KRTAP13
Pv86 KRTAP13
Pv96 KRTAP13
Pv68 KRTAP13
Pv71 KRTAP13
0.1

Figure 17

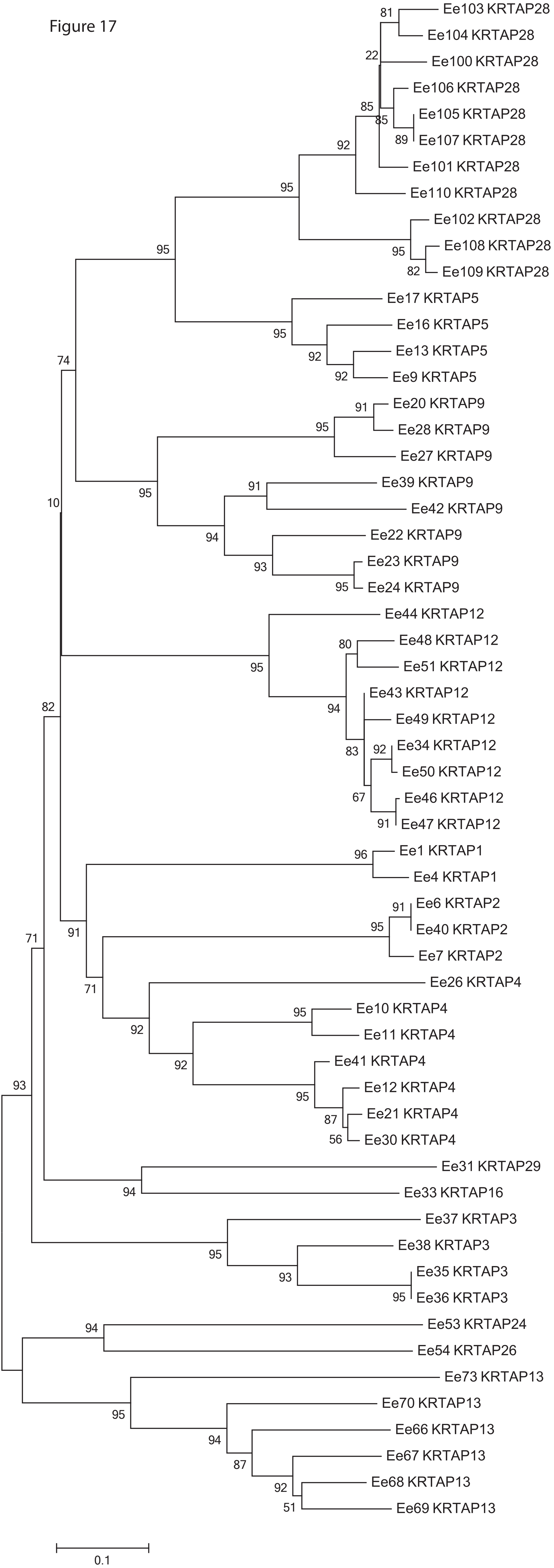
Ee103 KRTAP28
Ee104 KRTAP28
Ee100 KRTAP28
Ee106 KRTAP28
Ee105 KRTAP28
Ee107 KRTAP28
Ee101 KRTAP28
Ee110 KRTAP28
Ee102 KRTAP28
Ee108 KRTAP28
Ee109 KRTAP28
Ee17 KRTAP5
Ee16 KRTAP5
Ee13 KRTAP5
Ee9 KRTAP5
Ee20 KRTAP9
Ee28 KRTAP9
Ee27 KRTAP9
Ee39 KRTAP9
Ee42 KRTAP9
Ee22 KRTAP9
Ee23 KRTAP9
Ee24 KRTAP9
Ee44 KRTAP12
Ee48 KRTAP12
Ee51 KRTAP12
Ee43 KRTAP12
Ee49 KRTAP12
Ee34 KRTAP12
Ee50 KRTAP12
Ee46 KRTAP12
Ee47 KRTAP12
Ee1 KRTAP1
Ee4 KRTAP1
Ee6 KRTAP2
Ee40 KRTAP2
Ee7 KRTAP2
Ee26 KRTAP4
Ee10 KRTAP4
Ee11 KRTAP4
Ee41 KRTAP4
Ee12 KRTAP4
Ee21 KRTAP4
Ee30 KRTAP4
Ee31 KRTAP29
Ee33 KRTAP16
Ee37 KRTAP3
Ee38 KRTAP3
Ee35 KRTAP3
Ee36 KRTAP3
Ee53 KRTAP24
Ee54 KRTAP26
Ee73 KRTAP13
Ee70 KRTAP13
Ee66 KRTAP13
Ee67 KRTAP13
Ee68 KRTAP13
Ee69 KRTAP13
0.1

Figure 18

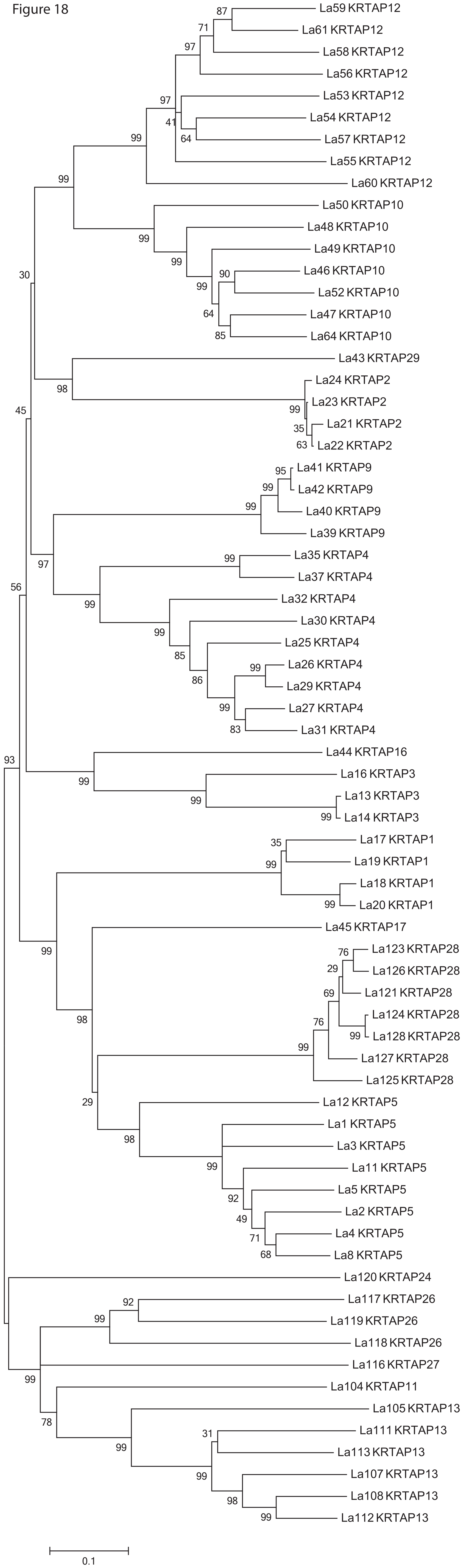
La59 KRTAP12
La61 KRTAP12
La58 KRTAP12
La56 KRTAP12
La53 KRTAP12
La54 KRTAP12
La57 KRTAP12
La55 KRTAP12
La60 KRTAP12
La50 KRTAP10
La48 KRTAP10
La49 KRTAP10
La46 KRTAP10
La52 KRTAP10
La47 KRTAP10
La64 KRTAP10
La43 KRTAP29
La24 KRTAP2
La23 KRTAP2
La21 KRTAP2
La22 KRTAP2
La41 KRTAP9
La42 KRTAP9
La40 KRTAP9
La39 KRTAP9
La35 KRTAP4
La37 KRTAP4
La32 KRTAP4
La30 KRTAP4
La25 KRTAP4
La26 KRTAP4
La29 KRTAP4
La27 KRTAP4
La31 KRTAP4
La44 KRTAP16
La16 KRTAP3
La13 KRTAP3
La14 KRTAP3
La17 KRTAP1
La19 KRTAP1
La18 KRTAP1
La20 KRTAP1
La45 KRTAP17
La123 KRTAP28
La126 KRTAP28
La121 KRTAP28
La124 KRTAP28
La128 KRTAP28
La127 KRTAP28
La125 KRTAP28
La12 KRTAP5
La1 KRTAP5
La3 KRTAP5
La11 KRTAP5
La5 KRTAP5
La2 KRTAP5
La4 KRTAP5
La8 KRTAP5
La120 KRTAP24
La117 KRTAP26
La119 KRTAP26
La118 KRTAP26
La116 KRTAP27
La104 KRTAP11
La105 KRTAP13
La111 KRTAP13
La113 KRTAP13
La107 KRTAP13
La108 KRTAP13
La112 KRTAP13
0.1

Figure 19

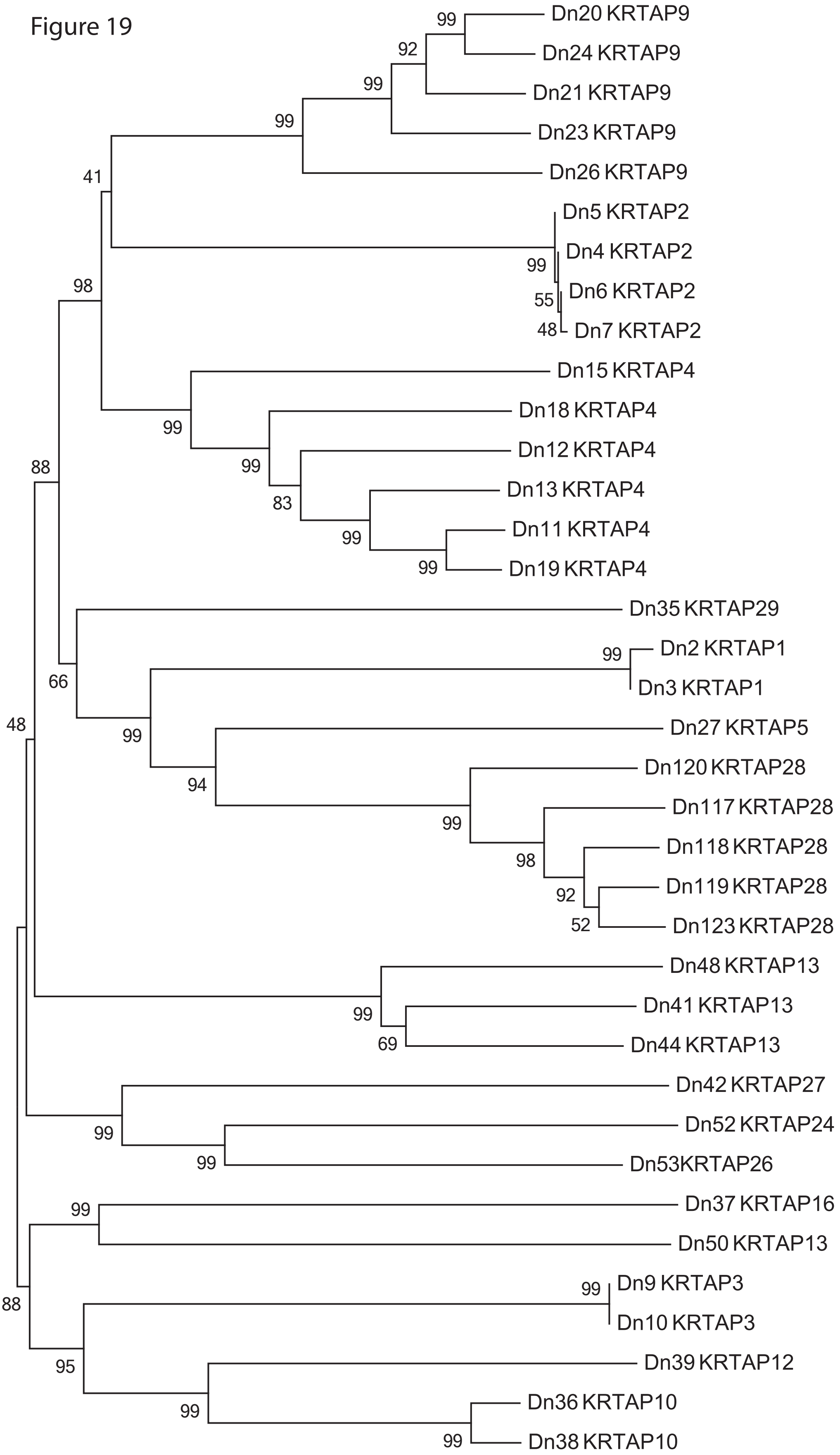

Dn20 KRTAP9
Dn24 KRTAP9
Dn21 KRTAP9
Dn23 KRTAP9
Dn26 KRTAP9
Dn5 KRTAP2
Dn4 KRTAP2
Dn6 KRTAP2
Dn7 KRTAP2
Dn15 KRTAP4
Dn18 KRTAP4
Dn12 KRTAP4
Dn13 KRTAP4
Dn11 KRTAP4
Dn19 KRTAP4
Dn35 KRTAP29
Dn2 KRTAP1
Dn3 KRTAP1
Dn27 KRTAP5
Dn120 KRTAP28
Dn117 KRTAP28
Dn118 KRTAP28
Dn119 KRTAP28
Dn123 KRTAP28
Dn48 KRTAP13
Dn41 KRTAP13
Dn44 KRTAP13
Dn42 KRTAP27
Dn52 KRTAP24
Dn53KRTAP26
Dn37 KRTAP16
Dn50 KRTAP13
Dn9 KRTAP3
Dn10 KRTAP3
Dn39 KRTAP12
Dn36 KRTAP10
Dn38 KRTAP10
99
92
99
99
41
99
55
48
98
99
99
83
99
99
88
66
99
99
48
94
99
98
92
52
99
69
99
99
99
99
88
95
99
99

Figure 20

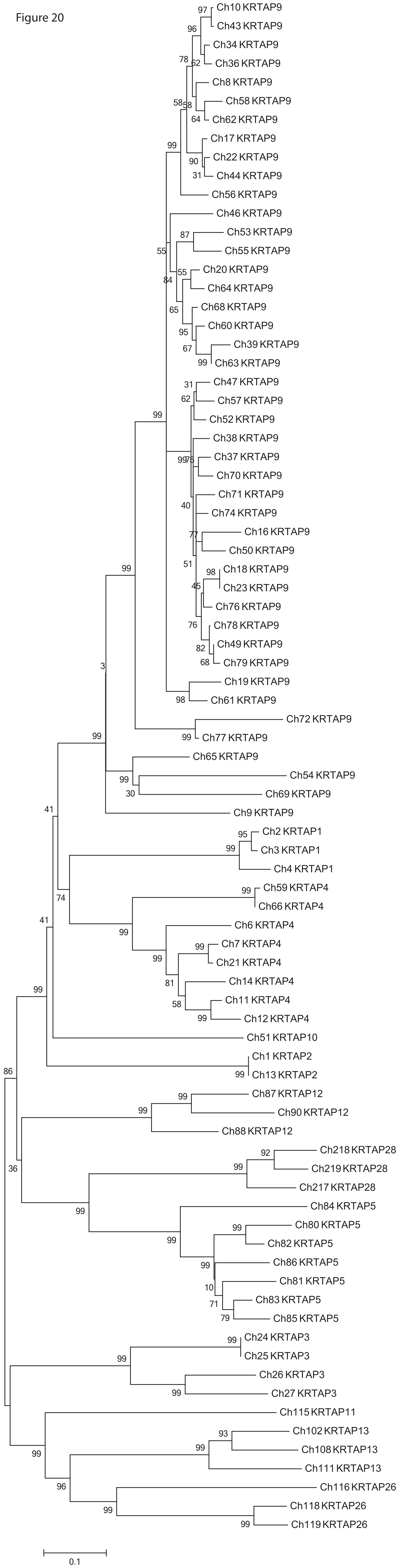
Ch10 KRTAP9
Ch43 KRTAP9
Ch34 KRTAP9
Ch36 KRTAP9
Ch8 KRTAP9
Ch58 KRTAP9
Ch62 KRTAP9
Ch17 KRTAP9
Ch22 KRTAP9
Ch44 KRTAP9
Ch56 KRTAP9
Ch46 KRTAP9
Ch53 KRTAP9
Ch55 KRTAP9
Ch20 KRTAP9
Ch64 KRTAP9
Ch68 KRTAP9
Ch60 KRTAP9
Ch39 KRTAP9
Ch63 KRTAP9
Ch47 KRTAP9
Ch57 KRTAP9
Ch52 KRTAP9
Ch38 KRTAP9
Ch37 KRTAP9
Ch70 KRTAP9
Ch71 KRTAP9
Ch74 KRTAP9
Ch16 KRTAP9
Ch50 KRTAP9
Ch18 KRTAP9
Ch23 KRTAP9
Ch76 KRTAP9
Ch78 KRTAP9
Ch49 KRTAP9
Ch79 KRTAP9
Ch19 KRTAP9
Ch61 KRTAP9
Ch72 KRTAP9
Ch77 KRTAP9
Ch65 KRTAP9
Ch54 KRTAP9
Ch69 KRTAP9
Ch9 KRTAP9
Ch2 KRTAP1
Ch3 KRTAP1
Ch4 KRTAP1
Ch59 KRTAP4
Ch66 KRTAP4
Ch6 KRTAP4
Ch7 KRTAP4
Ch21 KRTAP4
Ch14 KRTAP4
Ch11 KRTAP4
Ch12 KRTAP4
Ch51 KRTAP10
Ch1 KRTAP2
Ch13 KRTAP2
Ch87 KRTAP12
Ch90 KRTAP12
Ch88 KRTAP12
Ch218 KRTAP28
Ch219 KRTAP28
Ch217 KRTAP28
Ch84 KRTAP5
Ch80 KRTAP5
Ch82 KRTAP5
Ch86 KRTAP5
Ch81 KRTAP5
Ch83 KRTAP5
Ch85 KRTAP5
Ch24 KRTAP3
Ch25 KRTAP3
Ch26 KRTAP3
Ch27 KRTAP3
Ch115 KRTAP11
Ch102 KRTAP13
Ch108 KRTAP13
Ch111 KRTAP13
Ch116 KRTAP26
Ch118 KRTAP26
Ch119 KRTAP26
0.1

Figure 21

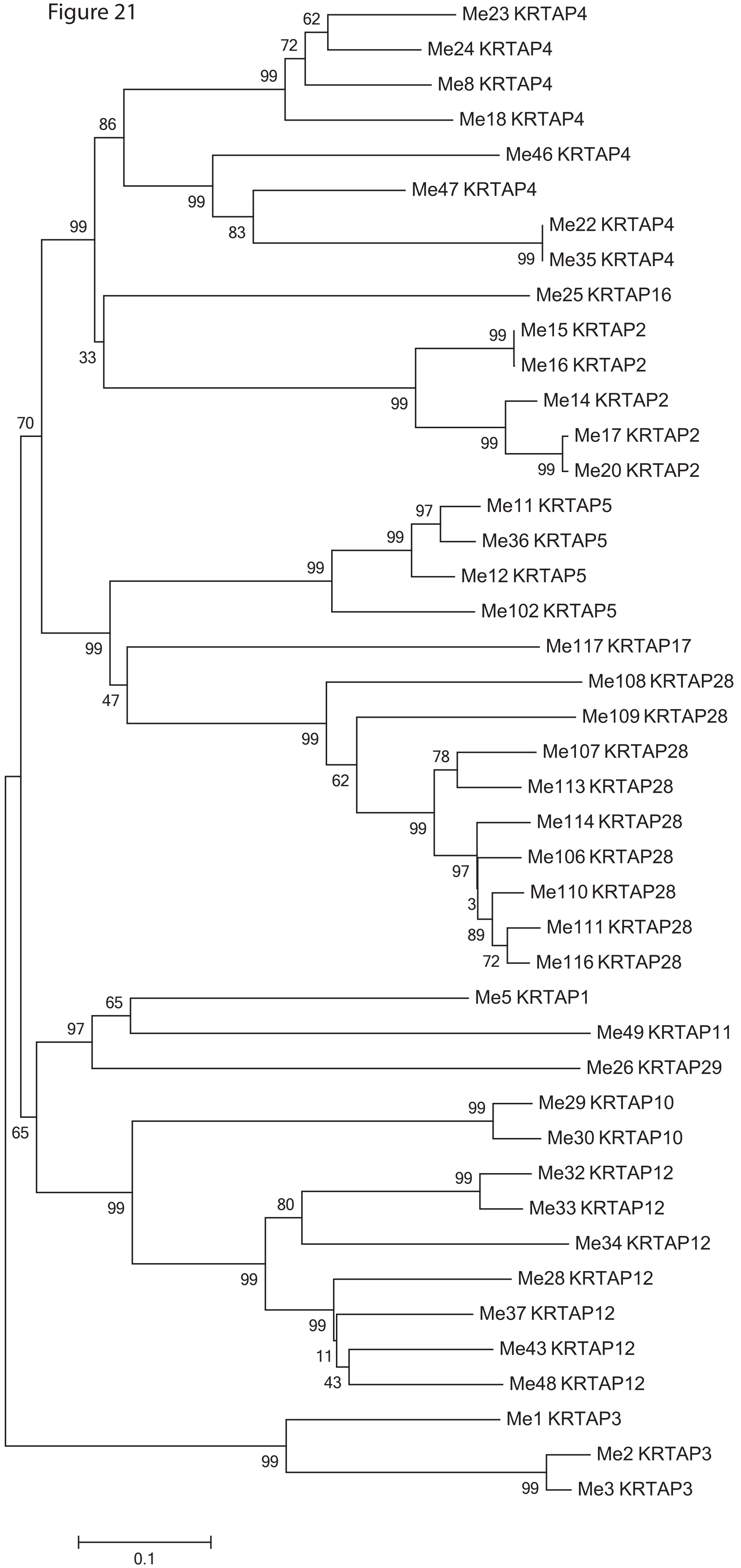

Me23 KRTAP4
Me24 KRTAP4
Me8 KRTAP4
Me18 KRTAP4
Me46 KRTAP4
Me47 KRTAP4
Me22 KRTAP4
Me35 KRTAP4
Me25 KRTAP16
Me15 KRTAP2
Me16 KRTAP2
Me14 KRTAP2
Me17 KRTAP2
Me20 KRTAP2
Me11 KRTAP5
Me36 KRTAP5
Me12 KRTAP5
Me102 KRTAP5
Me117 KRTAP17
Me108 KRTAP28
Me109 KRTAP28
Me107 KRTAP28
Me113 KRTAP28
Me114 KRTAP28
Me106 KRTAP28
Me110 KRTAP28
Me111 KRTAP28
Me116 KRTAP28
Me5 KRTAP1
Me49 KRTAP11
Me26 KRTAP29
Me29 KRTAP10
Me30 KRTAP10
Me32 KRTAP12
Me33 KRTAP12
Me34 KRTAP12
Me28 KRTAP12
Me37 KRTAP12
Me43 KRTAP12
Me48 KRTAP12
Me1 KRTAP3
Me2 KRTAP3
Me3 KRTAP3
0.1

Figure 22

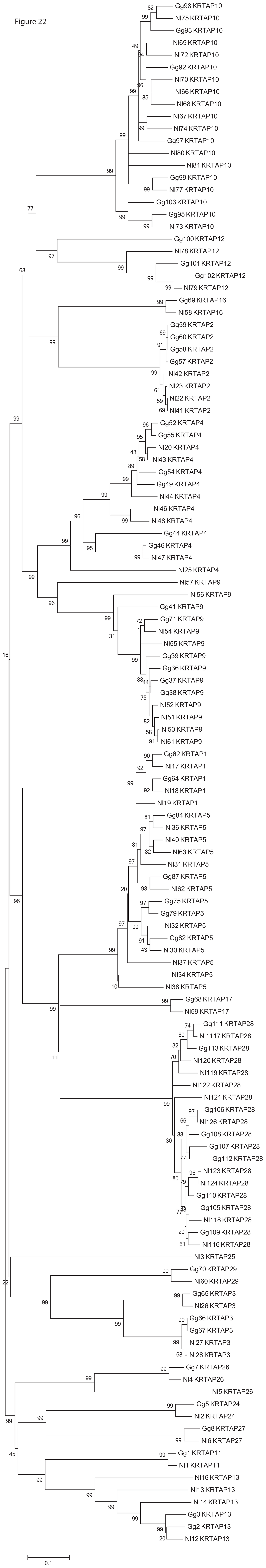
Gg98 KRTAP10
Nl75 KRTAP10
Gg93 KRTAP10
Nl69 KRTAP10
Nl72 KRTAP10
Gg92 KRTAP10
Nl70 KRTAP10
Nl66 KRTAP10
Nl68 KRTAP10
Nl67 KRTAP10
Nl74 KRTAP10
Gg97 KRTAP10
Nl80 KRTAP10
Nl81 KRTAP10
Gg99 KRTAP10
Nl77 KRTAP10
Gg103 KRTAP10
Gg95 KRTAP10
Nl73 KRTAP10
Gg100 KRTAP12
Nl78 KRTAP12
Gg101 KRTAP12
Gg102 KRTAP12
Nl79 KRTAP12
Gg69 KRTAP16
Nl58 KRTAP16
Gg59 KRTAP2
Gg60 KRTAP2
Gg58 KRTAP2
Gg57 KRTAP2
Nl42 KRTAP2
Nl23 KRTAP2
Nl22 KRTAP2
Nl41 KRTAP2
Gg52 KRTAP4
Gg55 KRTAP4
Nl20 KRTAP4
Nl43 KRTAP4
Gg54 KRTAP4
Gg49 KRTAP4
Nl44 KRTAP4
Nl46 KRTAP4
Nl48 KRTAP4
Gg44 KRTAP4
Gg46 KRTAP4
Nl47 KRTAP4
Nl25 KRTAP4
Nl57 KRTAP9
Nl56 KRTAP9
Gg41 KRTAP9
Gg71 KRTAP9
Nl54 KRTAP9
Nl55 KRTAP9
Gg39 KRTAP9
Gg36 KRTAP9
Gg37 KRTAP9
Gg38 KRTAP9
Nl52 KRTAP9
Nl51 KRTAP9
Nl50 KRTAP9
Nl61 KRTAP9
Gg62 KRTAP1
Nl17 KRTAP1
Gg64 KRTAP1
Nl18 KRTAP1
Nl19 KRTAP1
Gg84 KRTAP5
Nl36 KRTAP5
Nl40 KRTAP5
Nl63 KRTAP5
Nl31 KRTAP5
Gg87 KRTAP5
Nl62 KRTAP5
Gg75 KRTAP5
Gg79 KRTAP5
Nl32 KRTAP5
Gg82 KRTAP5
Nl30 KRTAP5
Nl37 KRTAP5
Nl34 KRTAP5
Nl38 KRTAP5
Gg68 KRTAP17
Nl59 KRTAP17
Gg111 KRTAP28
Nl1117 KRTAP28
Gg113 KRTAP28
Nl120 KRTAP28
Nl119 KRTAP28
Nl122 KRTAP28
Nl121 KRTAP28
Gg106 KRTAP28
Nl126 KRTAP28
Gg108 KRTAP28
Gg107 KRTAP28
Gg112 KRTAP28
Nl123 KRTAP28
Nl124 KRTAP28
Gg110 KRTAP28
Gg105 KRTAP28
Nl118 KRTAP28
Gg109 KRTAP28
Nl116 KRTAP28
Nl3 KRTAP25
Gg70 KRTAP29
Nl60 KRTAP29
Gg65 KRTAP3
Nl26 KRTAP3
Gg66 KRTAP3
Gg67 KRTAP3
Nl27 KRTAP3
Nl28 KRTAP3
Gg7 KRTAP26
Nl4 KRTAP26
Nl5 KRTAP26
Gg5 KRTAP24
Nl2 KRTAP24
Gg8 KRTAP27
Nl6 KRTAP27
Gg1 KRTAP11
Nl1 KRTAP11
Nl16 KRTAP13
Nl13 KRTAP13
Nl14 KRTAP13
Gg3 KRTAP13
Gg2 KRTAP13
Nl12 KRTAP13
0.1

Figure 23

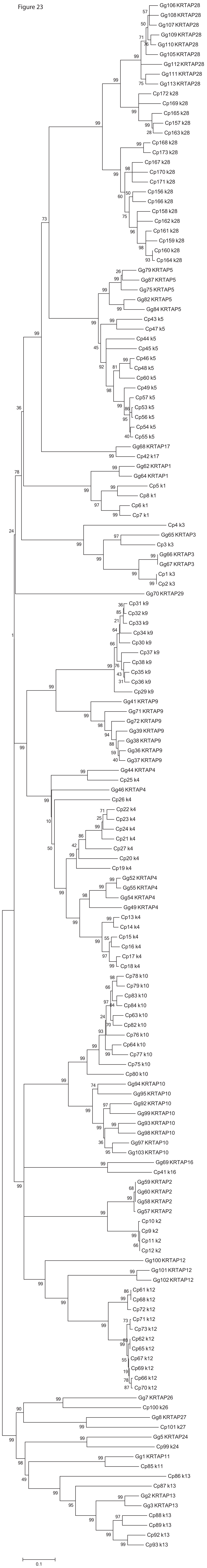

Gg106 KRTAP28
Gg108 KRTAP28
Gg107 KRTAP28
Gg109 KRTAP28
Gg110 KRTAP28
Gg105 KRTAP28
Gg112 KRTAP28
Gg111 KRTAP28
Gg113 KRTAP28
Cp172 k28
Cp169 k28
Cp165 k28
Cp157 k28
Cp163 k28
Cp168 k28
Cp173 k28
Cp167 k28
Cp170 k28
Cp171 k28
Cp156 k28
Cp166 k28
Cp158 k28
Cp162 k28
Cp161 k28
Cp159 k28
Cp160 k28
Cp164 k28
Gg79 KRTAP5
Gg87 KRTAP5
Gg75 KRTAP5
Gg82 KRTAP5
Gg84 KRTAP5
Cp43 k5
Cp47 k5
Cp44 k5
Cp45 k5
Cp46 k5
Cp48 k5
Cp60 k5
Cp49 k5
Cp57 k5
Cp53 k5
Cp56 k5
Cp54 k5
Cp55 k5
Gg68 KRTAP17
Cp42 k17
Gg62 KRTAP1
Gg64 KRTAP1
Cp5 k1
Cp8 k1
Cp6 k1
Cp7 k1
Cp4 k3
Gg65 KRTAP3
Cp3 k3
Gg66 KRTAP3
Gg67 KRTAP3
Cp1 k3
Cp2 k3
Gg70 KRTAP29
Cp31 k9
Cp32 k9
Cp33 k9
Cp34 k9
Cp30 k9
Cp37 k9
Cp38 k9
Cp35 k9
Cp36 k9
Cp29 k9
Gg41 KRTAP9
Gg71 KRTAP9
Gg72 KRTAP9
Gg39 KRTAP9
Gg38 KRTAP9
Gg36 KRTAP9
Gg37 KRTAP9
Gg44 KRTAP4
Cp25 k4
Gg46 KRTAP4
Cp26 k4
Cp22 k4
Cp23 k4
Cp24 k4
Cp21 k4
Cp27 k4
Cp20 k4
Cp19 k4
Gg52 KRTAP4
Gg55 KRTAP4
Gg54 KRTAP4
Gg49 KRTAP4
Cp13 k4
Cp14 k4
Cp15 k4
Cp16 k4
Cp17 k4
Cp18 k4
Cp78 k10
Cp79 k10
Cp83 k10
Cp84 k10
Cp63 k10
Cp82 k10
Cp76 k10
Cp64 k10
Cp77 k10
Cp75 k10
Cp80 k10
Gg94 KRTAP10
Gg95 KRTAP10
Gg92 KRTAP10
Gg99 KRTAP10
Gg93 KRTAP10
Gg98 KRTAP10
Gg97 KRTAP10
Gg103 KRTAP10
Gg69 KRTAP16
Cp41 k16
Gg59 KRTAP2
Gg60 KRTAP2
Gg58 KRTAP2
Gg57 KRTAP2
Cp10 k2
Cp9 k2
Cp11 k2
Cp12 k2
Gg100 KRTAP12
Gg101 KRTAP12
Gg102 KRTAP12
Cp61 k12
Cp68 k12
Cp72 k12
Cp71 k12
Cp73 k12
Cp62 k12
Cp65 k12
Cp67 k12
Cp69 k12
Cp66 k12
Cp70 k12
Gg7 KRTAP26
Cp100 k26
Gg8 KRTAP27
Cp101 k27
Gg5 KRTAP24
Cp99 k24
Gg1 KRTAP11
Cp85 k11
Cp86 k13
Cp87 k13
Gg2 KRTAP13
Gg3 KRTAP13
Cp88 k13
Cp89 k13
Cp92 k13
Cp93 k13
0.1

Figure 24

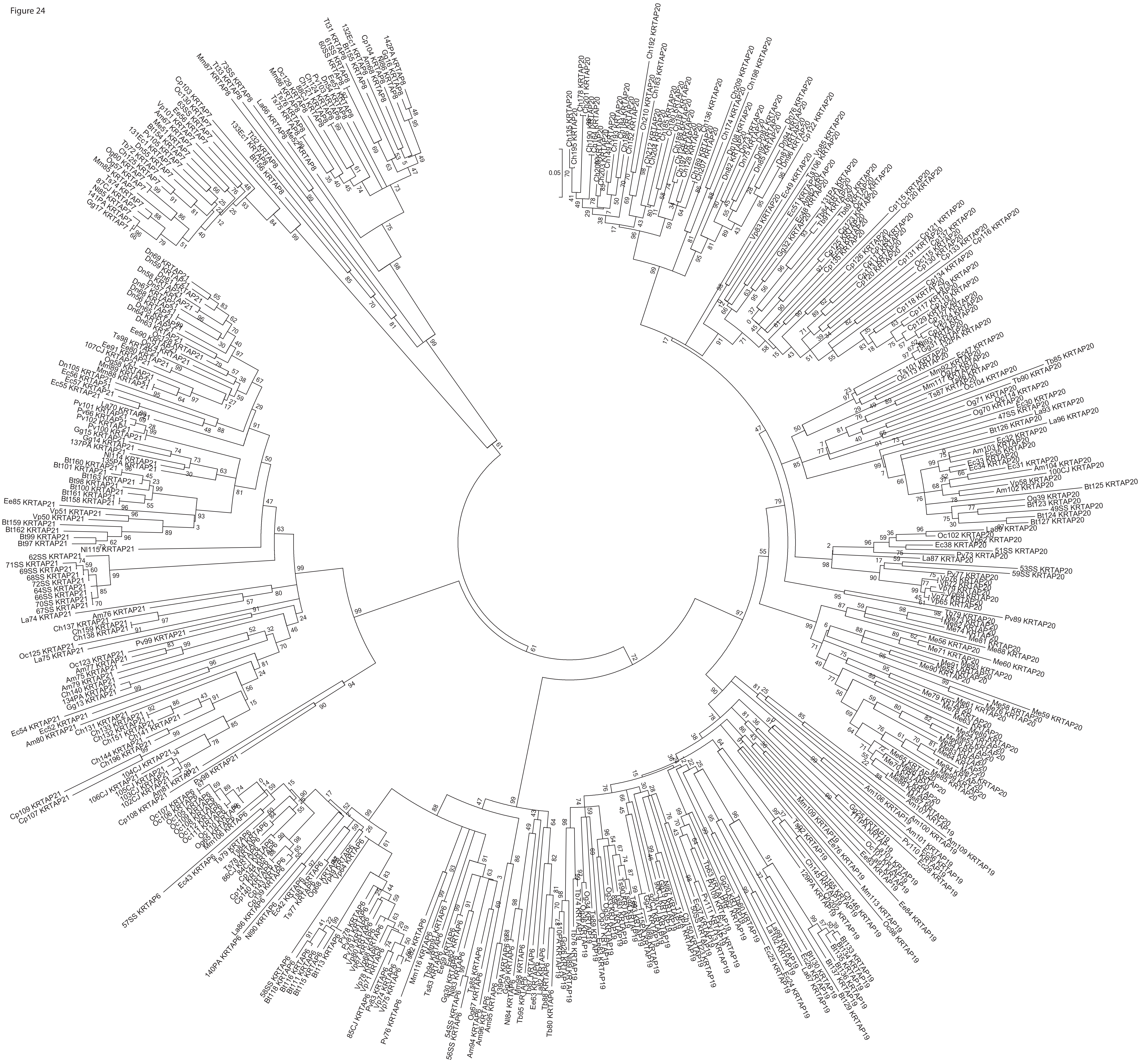

Supplement: Supplementary file 1 — Additional file 1: Figure S1-S24: The phylogeny of high cysteine KRTAP genes in 22 mammalian species. Neighbor-joining method with P-distance and interiors branch test with 1,000 replications (shown on the branches) was employed to build the trees. Figures S23 and S24 shows loss of, one to one orthologous relationship between two species due to concerted evolution. The KRTAP members are labeled with species abbreviation, Gene ID and KRTAP subfamily (Additional file 2) Figure S1-21 are in order, Gorilla, Pongo, Gibbon, Mormoset, Tarsies, Mouse lemur, Bushbaby, Treeshrew, Cavia, rabbit, Cow, Pig, Alpaca, Horse, Panda, Bat, Hedgehog, Elephant, Armadillo, Sloth and Wallaby. Figure S22 (Gorilla and Gibbon) and S23 (Gorilla and Cavia) shows reduced orthology with increase in divergence time. Figure S24 shows relationship between all HGT members in 22 genomes. (PDF 4 MB) [file 12864_2014_6484_MOESM1_ESM.pdf]
